# Anti-Proliferative Effect of Triterpenoidal Glycosides from the Roots of *Anemone vitifolia* through a Pro-Apoptotic Way

**DOI:** 10.3390/molecules22040642

**Published:** 2017-04-17

**Authors:** Changcai Bai, Yunyun Ye, Xiao Feng, Ruifeng Bai, Lu Han, Xiuping Zhou, Xinyao Yang, Pengfei Tu, Xingyun Chai

**Affiliations:** 1 Key Laboratory of Hui Medicine Modernization, Ministry of Education, Ningxia Medical University Pharmacy College, Yinchuan 750004, China; yeyunyun2016@163.com (Y.Y.); lulu2008han@163.com (L.H.); 18295498705@163.com (X.Z.); 2 Modern Research Center for Traditional Chinese Medicine, School of Chinese Materia Medica, Beijing University of Chinese Medicine, Beijing 100029, China; fengxiao931209@163.com (X.F.); bairuifeng0601@163.com (R.B.); 18811792712@163.com (X.Y.); pengfeitu@163.com (P.T.)

**Keywords:** *Anemone vitifolia*, cytotoxicity-guided fractionation, oleanane saponin, anti-proliferation, apoptosis, HepG2

## Abstract

A cytotoxicity-guided phytochemical investigation of *Anemone vitifolia* roots led to the isolation of six oleanane saponins (**1**–**6**), which were reported from the species for the first time. Their structures were determined by comparing its MS and NMR data with those in literature. Compounds **1**–**4** showed significant inhibitory effects on the proliferation of hepatocellular carcinoma HepG2 cells with IC_50_ values ranging from 2.0 to 8.5 μM, compared to positive control methotrexate with IC_50_ value of 15.8 μM. Flow cytometry analysis revealed that compounds **1**–**4** exerted anti-proliferative effects through a pro-apoptotic way of hepatocellular carcinoma cells.

## 1. Introduction

*Anemone* L. is a big genus in the family of Ranunculaceae, which consists of 150 species across the world [1]. As the major and characteristic constituents in this genus, oleanolic acid and hederagenin triterpenoid saponin showed anti-tumor, anti-inflammatory, and analgesic activities, etc. [2,3,4,5,6]. *A. vitifolia* Buch-Ham is one of the most representative medicinal plants, which is mainly distributed in northwestern and southwestern China, northern Burma, Bhutan, Sikkim, Nepal, and northern India [1]. Long-term practice and traditional use has proved its significant activity on the treatment of rheumatoid joint pain, enteritis, and diarrhea, in addition to its use in natural pesticides [7,8,9]. However, no phytochemical and pharmacological activity study of this botanical specimen has yet been performed.

A cytotoxicity-guided fractionation on the roots of *A. vitifolia* was conducted, which led to the isolation and identification of six triterpenoidal glycosides (**1**–**6**). The compounds showed significant inhibitory effects on cell proliferation of hepatocellular carcinoma with IC_50_ values ranging from 2.5 to 12.0 μM, compared to the positive control of methotrexate. Flow cytometry analysis revealed that compounds **1**–**4** remarkably induced apoptosis of HepG2 cells. Here in, this work documents the isolation, cytotoxic evaluation and apoptotic analysis of these constituents from *A. vitifolia*.

## 2. Results and Discussion

### 2.1. Structural Identification of Compounds ***1***–***6***

**Figure 1:**
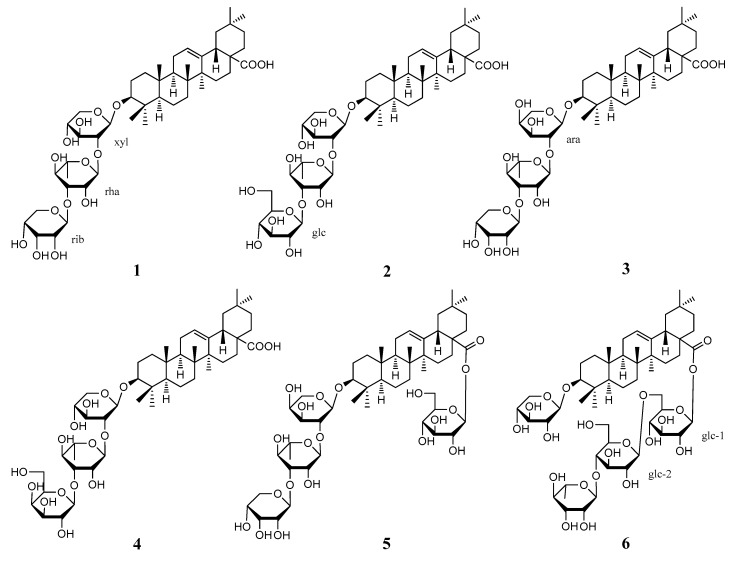
Structures of **1**–**6** from the roots of *A. vitifolia*.

Although *A. vitifolia* is traditionally used for the treatment of rheumatoid arthritis, no in vitro anti-inflammatory activity was observed in our previous study of this specimen. Inspired by *Anemone* saponins with significant antitumor activities reported in References [10,11,12,13], *A. vitifolia* was chemically investigated by a bioassay-guided separation against HepG2 cell lines in this study, which led to the discovery of the cytotoxic butanol soluble extract, fraction ABB, and six compounds (**1**–**6**). The chemical structures of the abovementioned compounds are shown in Figure 1.

3-*O*-β-d-Ribopyranosyl-(1→3)-*α*-l-rhamnopyranosyl-(1→2)-β-d-xylopyranosyl oleanolic acid (**1**) was obtained as white amorphous power with its specific rotation value of [α]${}_{D}^{25}$ −20.5 (*c* 0.1, MeOH). The ^1^H NMR (CD_3_OD, 500 MHz) data (Table S1) showed seven methyl signals at δ_H_ 0.81 (3H, s, Me-26), δ_H_ 0.86 (3H, s, Me-24), δ_H_ 0.91 (3H, s, Me-30), δ_H_ 0.94 (3H, s, Me-25, 29), δ_H_ 1.06 (3H, s, Me-23), and 1.16 (3H, s, Me-27), and one olefinic proton at δ_H_ 5.24 (1H, br. s, H-12). Based on the above results, and combined with the ^13^C NMR (CD_3_OD, 125 MHz) data (Table S1), compound **1** is suggested to be an oleanane type triterpenoid. Other ^1^H NMR data of δ_H_ 1.23 (3H, d, *J* = 6.0 Hz, Me of rhamnose), 3.13 (1H, dd, *J* = 11.5, 4.0 Hz, H-3), δ_H_ 4.38 (1H, d, *J* = 7.0 Hz, H-1 of xylose), δ_H_ 4.99 (1H, d, *J* = 4.0 Hz, H-1 of ribose), and δ_H_ 5.36 (1H, s, H-1 of rhamnose) suggested the presence of sugar moieties in the molecule, which was proved by HPLC analysis after acid hydrolysis [14]. Comparison of all the spectroscopic data showed agreement with those in the literature, and the structure of **1** was therefore determined [15].

3-*O*-β-d-Glucopyranosyl-(1→3)-*α*-l-rhamnopyranosyl-(1→2)-β-d-xylopyranosyl oleanolic acid (**2**) was obtained as white amorphous power ([α${]}_{D}^{25}$ −3.5 (*c* 0.01, MeOH)). Its molecular formula was determined as C_47_H_74_O_16_ by HR-ESI-MS data at *m/z*: [M − H]^−^ 895.5047 (calcd. for C_47_H_76_O_16_ 895.5061). The ^1^H and ^13^C NMR data (Table S1) showed the presence of seven methyl signals at δ_H_ 0.81 (3H, s, Me-26), δ_H_ 0.86 (3H, s, Me-24), δ_H_ 0.91 (3H, s, Me-30), δ_H_ 0.94 (6H, s, Me-25, 29), δ_H_ 1.06 (3H, s, Me-23), and 1.17 (3H, s, Me-27), one olefinic proton at δ_H_ 5.24 (1H, br. s, H-12), and sugar moiety, suggesting a similar structure to that of **1**, except for the outer sugar. Detailed NMR data comparison of **2** with those previously reported [16] identified the structure.

3-*O*-β-d-Ribopyranosyl-(1→3)-*α*-l-rhamnopyranosyl-(1→2)-*α*-l-arabinopyranosyl oleanolic acid (**3**) was obtained as white amorphous power with its specific rotation value of [α${]}_{D}^{25}$ −22.8 (*c* 0.1, MeOH). Its molecular formula C_46_H_74_O_15_ was defined by HR-ESI-MS data at *m*/*z*: [M − H]^−^ 865.4913 (calcd. for C_46_H_74_O_15_ 865.4955) and ^13^C NMR data. The ^1^H and ^13^C NMR data (CD_3_OD) (Table S1) showed the presence of seven methyl signals, which included one olefin, one carboxyl, and three sugar units, suggesting a similar structure to those of **1** and **2**. Finally, by comparing all physiochemical data with those reported [17,18,19], the structure of **3** was assigned.

3-*O*-β-d-Galactopyranosyl-(1→3)-*α*-l-rhamnopyranosyl-(1→2)-β-d-xylopyranosyl oleanolic acid (**4**) was obtained as white amorphous power with its specific rotation value of [α${]}_{D}^{25}$ −30.0 (*c* 0.1, MeOH). The ^1^H and ^13^ C NMR data (Table S2) is similar to those of **1** and **2**, suggesting their close structures, except for the outer sugar unit. The discrepancy was confirmed by HPLC determination after acid hydrolysis of **4**. Finally, by comparing all physiochemical data with those reported in the literature, the structure of compound **4** was determined [20,21].

Clematichinenoside A (**5**) was obtained as white amorphous power. Its molecular formula C_52_H_84_O_20_ was determined by HR-ESI-MS data at *m*/*z*: [M + Na]^+^ 1051.5446 (calcd. for C_52_H_84_O_20_Na, 1051.5454). The ^1^H NMR (C_5_D_5_N, 500 MHz) data showed the presence of seven methyl signals, one olefinic proton, and four anomeric protons at δ_H_ 4.86 (1H, d, *J* = 5.0 Hz, H-1 of arabinose), δ_H_ 5.99 (1H, d, *J* = 4.5 Hz, H-1 of ribose), δ_H_6.36 (1H, d, *J* = 8.5, H-1 of glucose), δ_H_ 6.68 (1H, s, H-1 of rhamnose), which were supported by analysis of its ^13^C NMR (C_5_D_5_N, 125 MHz) data (Table S2). A comparison of the NMR data with that of **3** suggested its structural similarity, except for an additional glycosylation at C-28. All of the above data is in good agreement with that previously reported [22], thus the structure of **5** was determined.

Anhuienoside C (**6**) was obtained as white amorphous powder. Its molecular formula C_53_H_86_O_21_ was determined by analysis of HR-ESI-MS data at *m/z*: [M − H]^−^ 1057.5395 (calcd. for C_53_H_86_O_21_ 1057.5589). The ^1^H and ^13^C NMR data (Table S2) data suggested the presence of an oleanane aglycone, including the representative seven methyls, one olefin, and a carboxyl, as well as four anomeric protons at δ_H_4.81 (1H, d, *J* = 7.5 Hz, H-1 of xylose), δ_H_ 5.00 (1H, d, *J* = 8.0 Hz, H-1 of glucose-2), δ_H_ 5.86 (1H, br. s, H-1 of rhamnose), and δ_H_ 6.25 (1H, d, *J* = 8.0 Hz, H-1 of glucose-1). A comparison of the ^1^H and ^13^C NMR data with that reported in literature revealed their good accordance, and the structure of **6** was therefore determined [22].

### 2.2. Inhibitory Effect on Proliferation of HepG2 Cells

**Figure 2:**
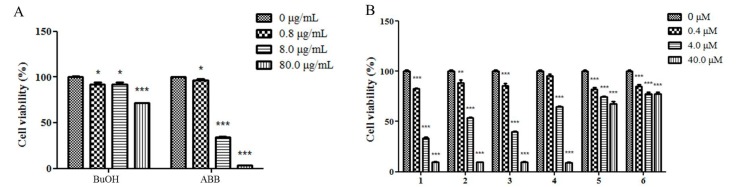
The inhibitory effects of butanol (BuOH) extract, fraction ABB and compounds **1**–**6** on cell proliferation of HepG2 cells. (**A**) HepG2 cells treated with BuOH extract and ABB fraction at the concentrations of 0, 0.8, 8.0, and 80.0 μg/mL for 48 h were subjected to cell viability assay. * *p* < 0.05, ** *p* < 0.01, and *** *p* < 0.001; (**B**) HepG2 cells treated with **1**–**6** at the concentrations of 0, 0.4, 4.0, and 40.0 μM, respectively. After incubation for 48 h, cells were subjected to cell viability assay. * *p* < 0.05, ** *p* < 0.01 and *** *p* < 0.001.

A cytotoxicity evaluation against the growth of HepG2 cells was performed on the butanol (BuOH) soluble extract, ethyl acetate (EtOAc) soluble extract, and petroleum ether (PE) soluble extract, as well as two fractions ABB and ABC at 0.8, 8.0, and 80.0 μg/mL, and six isolates at 0.4, 4.0, and 40.0 μM. The results showed that the BuOH soluble extract significantly inhibited the cell viability of HepG2 cells at 80.0 μg/mL. The fraction ABB exhibited a remarkable inhibitory effect on proliferation of HepG2 cells at 0.8–8.0 μg/mL (Figure 2A), whereas no obvious inhibition of the PE extract, EtOAc extract, and ABC fractions against HepG2 cells was observed. Furthermore, compounds **1**–**4** exhibited remarkable cytotoxicity at 0.4–4.0 μM compared with methotrexate (the positive control) with an IC_50_ value of 15.8 μM, whereas **5** and **6** were less cytotoxic (Figure 2B). Further evaluation showed that the IC_50_ values of **1**–**4** were 2.0, 5.3, 3.2, and 8.5 μM, respectively, and the IC_50_ value of ABB was 11.7 μg/mL. The above data demonstrated that ABB and compounds **1**–**4** contributed to the cytotoxicity of *A. vitifolia.*

### 2.3. Flow Cytometry Analysis

**Figure 3:**
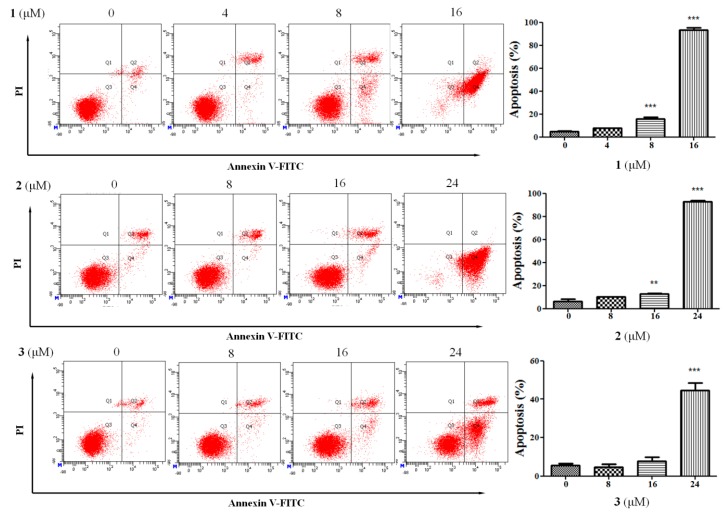

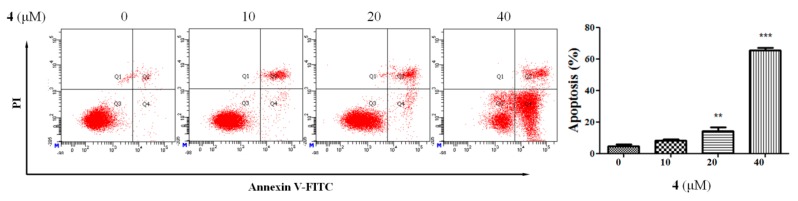
Compounds **1**–**4** induced apoptosis in HepG2 cells. HepG2 cells treated with **1**–**4** in different concentrations, respectively, were subjected to apoptosis by flow cytometry after 48 h of drug incubation. Annexin V-FITC positive/PI negative cells were regarded as early apoptotic, while Annexin V-FITC negative/PI positive cells were regarded as late apoptotic. Data are presented as the mean ± SEM, *n* = 3, ** *p* < 0.01, *** *p* < 0.001.

Apoptosis is an important physiological mechanism of cell death. Inhibition of normal cell apoptosis in the body could led to proliferative diseases, such as tumors and autoimmune diseases. Therefore, the modulation of apoptosis may be a feasible means for the prevention and treatment of these diseases, which is an effective way for the discovery of leading compounds [23,24,25,26]. Flow cytometry analysis with Annexin V-FITC/propidium iodide (PI) is widely used to detect apoptosis. Therefore, the effects of **1**–**4** on the apoptosis of HepG2 cells were examined using flow cytometry analysis. As shown in Figure 3, these compounds induced apoptosis of HepG2 cells, especially at high concentrations. Moreover, most of the staining cells were in the Q_4_ field of flow cytometry chart after treatment of **1**–**4** for 48 h, suggesting that **1**–**4** induced apoptosis of HepG2 cells mainly at the early stage. Taken together, these saponins exerted anti-proliferative effect through triggering apoptosis of hepatocellular carcinoma cells.

### 2.4. Preliminary Structure-Activity Relationship Analysis

**Figure 4:**
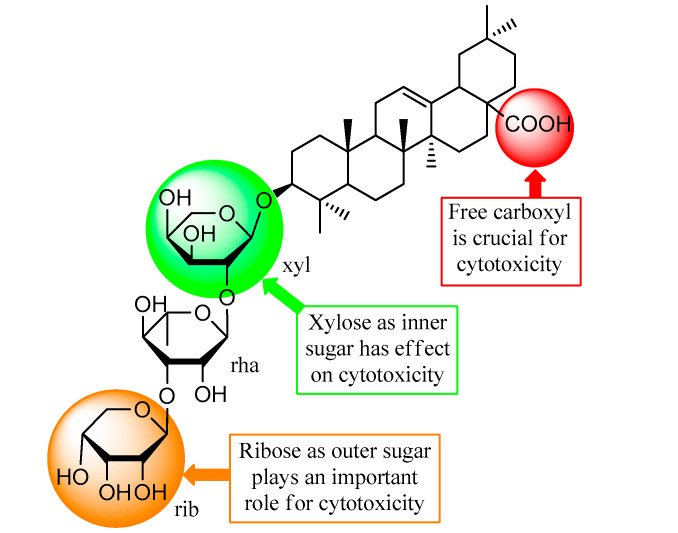
Brief structure-activity relationship analysis of the *Anemone* saponins.

A brief structure-activity relationship (SAR) analysis (Figure 4) of compounds **1**–**6** inferred that the presence of free C-28 carboxyl functionality is crucial to the cytotoxicity against HepG2 cells. This is the reason why compounds **1**–**4** were significantly active rather than **5** or **6**. SAR analysis of **1**–**4** indicated that the ribose as the outer sugar might play an important role, and the xylose as the inner sugar also has considerable impact on the activity. However, this is only a preliminary SAR result, and more samples are required for a substantial conclusion.

## 3. Experimental Section

### 3.1. General Procedure

NMR spectra were measured on a Varian-500 spectrometer (Varian Inc., Palo Alto, CA, USA). HR-ESI-MS were recorded using a Shimadzu LC-MS-IT-TOF (Shimadzu, Tokyo, Japan). Optical rotations were measured on a Rudolph Autopol IV automatic polarimeter (Rudolph Research Analytical). IR spectra were recorded on a Thermo Nicolet Nexus 470 FT-IR spectrophotometer with KBr pellets (Nicolet Company, Madison, WI, USA). Preparative high-performance liquid chromatography (HPLC) was performed by using a Waters 2535 pump system (Waters corporation, Milford, MA, USA) equipped with YMC-Pack C_18_ (250 × 10 mm, 5 μm). HPLC was performed on a Shimadzu LC-20A pump system (Shimadzu Corporation, Tokyo, Japan), equipped with an SPD-M20A photodiode array detector monitoring, and with an analytical RP-HPLC column (Agilent XDB-C_18_, 250 × 4.6 mm, 5 μm). Silica gel (200‒300 mesh, Qingdao Haiyang Chem. Co. Ltd., Qingdao, China), Sephadex LH-20 (GE Healthcare, Umea, Sweden), and LiChroprep RP-18 (Merck, 40–63 μm, Darmstadt, Germany) were used for column chromatography (CC). Thin layer chromatography (TLC) was carried out on pre-coated silica gel GF254 (Qingdao, Haiyang Chem. Co. Ltd., Qingdao, China) and RP-18F254S (Merck, Germany) plates.

### 3.2. Plant Material

Roots of *A. vitifolia* were collected by Changcai Bai from Huan County, Gansu Province, China, in July 2012. The plant material was authenticated as the *A. vitifolia* Buch-Ham. by Professor Zhigang Ma (Lanzhou University Pharmacy College, Lanzhou, China). A voucher specimen (AV201207) has been deposited in the herbarium of Ningxia Medical University Pharmacy College, Yinchuan, China.

### 3.3. Isolation of Compounds

The dried roots of *A. vitifolia* (4 kg) were extracted with 70% EtOH under reflux three separate times. After the removal of solvents under reduced pressure, the residue (1.2 kg) was suspended in water (4 L) and extracted with petroleum ether (PE) (3 × 5 L), ethyl acetate (EtOAc) (3 × 5 L), and butanol (BuOH) (3 × 5 L). The BuOH soluble extract (300 g) was subjected to silica gel column chromatography (CC) with a gradient of CH_2_Cl_2_-MeOH (from 5:2 to 0:100, 5% formic acid) to give two major fractions, ABB and ABC, which were screened for cytotoxicity.

The cytotoxic Fr. ABB (18 g) was subjected to silica gel CC with a gradient elution with EtOAc-MeOH (10:1–0:1, 5% formic acid) to give nine fractions (B1–B9). B8 was subjected to Sephadex LH-20 CC eluting with MeOH to afford four subfractions (B8a–B8d). B8c (74 mg) was isolated with ODS CC by eluting with MeOH–H_2_O (1:1–1:0) to give three fractions (B8c1–B8c2), and B8c2 was further separated by semi-preparative HPLC with the detection wavelength of 210 nm, flow rate of 3.0 mL/min, and mobile phase of MeOH/H_2_O (82:18) to provide **1** (30.1 mg, *t*_R_ = 35.3 min). B8b (480 mg) was separated by an opening ODS CC, eluting with MeOH–H_2_O (1:1–1:0), followed by purification on semi-preparative HPLC (MeOH–H_2_O 82:18) to afford **3** (7.0 mg, *t*_R_ = 43.7 min) and **2** (21.0 mg, *t*_R_ = 45.9 min). B4 (600 mg) was subjected to Sephadex LH-20 CC (MeOH) to afford three subfractions B4a–B4c. The main B4a was subjected to silica gel CC, eluting with a gradient CH_2_Cl_2_–MeOH (1:1), followed by purification with ODS CC (MeOH–H_2_O 1:4–1:0) to give **4** (13.2 mg). B6 was subjected to Sephadex LH-20 CC (MeOH), then the major subfraction B6a (350 mg) was further separated by semi-preparative HPLC (MeOH–H_2_O 76:24) to provide **5** (14.8 mg, *t*_R_ = 26.8 min). B7 was subjected to Sephadex LH-20 CC to afford the main portion of B7a (510 mg), which was further chromatographed with an opening ODS column (MeOH–H_2_O 1:1–1:0) to obtain the subfraction B7a1. An HPLC purification of B7a1 by MeOH–H_2_O (75:25) afforded **6** (19.5 mg, *t*_R_ = 17.8 min).

### 3.4. Cytotoxicity Assay

The extracts, fractions and isolated compounds were dissolved in DMSO (Sigma, St. Louis, MO, USA, WSBB5403V, purity >99% by HPLC) as stock solutions. Before each bioassay, all of the stock solutions were diluted with DMEM (cellgro, 1 × with 4.5 g/L glucose, L-glutamine & sodium pyruvate, Manassas, VA, USA) to give final concentrations of 0.4, 4 and 40 μM containing less than 0.1% DMSO. HepG2 cells were seeded in 96-well plates at 3.5 × 10^3^ cells/well and incubated for 24 h. After that, the extracts, fractions and isolated compounds were added as described above for another 48-h incubation. In the meantime, methotrexate was used as a positive control, and during each bioassay 100 μL MTT (0.5 mg/mL) was added into each well for 4 h of incubation. Following that, the supernatant in each well was thrown and 150 μL DMSO was added. The plates were swiftly shaken to fully dissolve crystals, and then transferred to a microplate reader to measure the optical density at a wavelength of 570 nm.

### 3.5. Flow Cytometry Analysis

HepG2 cells were seeded in 12-well plates at a density of 4 × 10^4^ cells/well and were treated with **1**–**4**. Drugs with four concentrations were added to each plate, and each concentration was added to three wells for repetition. After treatment of drugs for 48 h, cells were collected and a reagent was added in Annexin V-FITC apoptosis detection kit (BD Pharmingen), according to the manufacturer’s instructions. Furthermore, early apoptotic cells were prominently stained by Annexin V-FITC, while late apoptotic cells were stained by PI (propidium iodide).

### 3.6. Data Analysis

The data are expressed as the mean ± the standard error of the mean (SEM), using Graph Pad Prism 5 and the Statistical Package for the Social Sciences (SPSS) 20.0 software. The differences of means of the measured parameters were compared by using one-way analysis of variance (ANOVA). The *p*-values < 0.01 were regarded as significant.

## 4. Conclusions

A cytotoxicity-guided fractionation on the roots of *Anemone vitifolia* led to the isolation of six oleanane saponins. Four of them (**1**–**4**) showed significant inhibition on the cell proliferation of hepatocellular carcinoma HepG2 cells with IC_50_ values of 2.0–8.5 μM, compared to positive control methotrexate with an IC_50_ value of 15.8 μM. Flow cytometry analyses revealed that these saponins exert the in vitro cytotoxic effect by remarkably inducing apoptosis of HepG2 cells, and a preliminary SAR analysis suggests that the free carboxyl in the molecules plays a vital role in this kind of biological activity. This paper represents the phytochemical investigation of the species, as well as the inhibitory effect of compounds **2**, **4**, and **6** on the proliferation of HepG2 cells, for the first time.

The present findings will not only enrich the discovery of cytotoxic leads for liver disease drug development, but also provide a reference for oleanane saponins research, especially for its SAR analysis.

## Supplementary Materials

Supplementary materials are available online. HRESIMS and ^1^H and ^13^C NMR spectroscopic spectra of compounds **1**–**6** and separation flow.
