# Supplementary material for: Anti-Proliferative Effect of Triterpenoidal Glycosides from the Roots of Anemone vitifolia through a Pro-Apoptotic Way
Source: Molecules. 2017 Apr 17;22(4):642. doi: 10.3390/molecules22040642 (PMC6154594; doi:10.3390/molecules22040642)
Supplement: Supplementary file 1 [file molecules-22-00642-s001.pdf]

Supplementary data

Anti-proliferative effect of triterpenoidal glycosides from the roots of *Anemone vitifolia* through regulating apoptosis-associated proteins

Changcai Bai [1, †, *], Yunyun Ye [1, 2, †], Xiao Feng [2, †], Ruifeng Bai [2], Lu Han [1], Xiuping Zhou [1], Xinyao Yang [2], Pengfei Tu [2], and Xingyun Chai [2, *]

[1]*Ningxia Medical University Pharmacy College, Key Laboratory of Hui Medicine Modernization, Ministry of Education, Yinchuan 750004, P. R. China;*

[2] *Modern Research Center for Traditional Chinese Medicine, School of Chinese Materia Medica, Beijing University of Chinese Medicine, Beijing 100029, P. R. China*

[†] These authors contribute to the paper equally.

[*]Corresponding authors:

Tel/fax: 86 951 6880 582, *E-mail address*: changcaibai@163.com (Changcai Bai)

Tel/fax: 86 10 6428 6350, *E-mail address*: xingyunchai@yeah.net (Xingyun Chai)

Table S1 ^{1}H and ^{13}C NMR (500/125 MHz) data of 1–3, δ in ppm, J in Hz

Table S2 ^{1}H and ^{13}C NMR (500/125 MHz) data of 4-6, δ in ppm, J in Hz

Figure S1 Analysis of the ratio of bax/bcl-2 in compounds 1 and 2 treatment groups

Figure S2 HPLC-PDA (210 nm) profiles of **1**

Figure S3 ^{1}H NMR spectrum of **1** in CD_3OD (500 MHz)

Figure S4 ^{13}C NMR spectrum of **1** in CD_3OD (125 MHz)

Figure S5 HPLC-PDA (210 nm) profiles of **2**

Figure S6 HR-ESI-MS spectrum of **2**

Figure S7 ^{1}H NMR spectrum of **2** in CD_3OD (500 MHz)

Figure S8 ^{13}C NMR spectrum of **2** in CD_3OD (125 MHz)

Figure S9 IR spectrum of **3**

Figure S10 HR-ESI-MS spectrum of **3**

Figure S11 HPLC-PDA (210 nm) profiles of **3**

Figure S12 ^{1}H NMR spectrum of **3** in CD_3OD (500 MHz)

Figure S13 ^{13}C NMR spectrum of **3** in CD_3OD (125 MHz)

Figure S14 ^{1}H-^{1}H COSY spectrum of **3**

Figure S15 HSQC spectrum of **3**

Figure S16 HMBC spectrum of **3**

Figure S17 general acid hydrolysis of **3**

Figure S18 HPLC-PDA (210 nm) profiles of **4**

Figure S19 ^{1}H NMR spectrum of **4** in Pyridine-d_5 (500 MHz)

Figure S20 ^{13}C NMR spectrum of **4** in Pyridine-d_5 (125 MHz)

Figure S21 ^{1}H NMR spectrum of **5** in Pyridine-d_5 (500 MHz)

Figure S22 ^{13}C NMR spectrum of **5** in Pyridine-d_5 (125 MHz)

Figure S23 HR-ESI-MS spectrum of **6**

Figure S24 ^{1}H NMR spectrum of **6** in Pyridine-d_5 (500 MHz)

Figure S25 ^{13}C NMR spectrum of **6** in Pyridine-d_5 (125 MHz)

Figure S26 The separation of the compounds 1-6

Table S1. ^{1}H and ^{13}C NMR (500/125 MHz) data of 1–3, δ in ppm, J in Hz

NO.	1		2		3	
	δ_C	δ_H	δ_C	δ_H	δ_C	δ_H
**1**	40.0	0.98 (m),1.61 (m)	40.0	0.98 (m), 1.59 (m)	40.0	0.99 (m), 1.60 (m)
**2**	27.3	1.67 (m),1.78 (m)	27.3	1.66 (m), 1.76 (m)	27.1	1.86 (m)
**3**	90.2	3.13 (dd, 4.0, 11.5)	90.2	3.11 (dd, 4.0, 11.5)	90.6	3.12 (dd, 4.0, 11.5)
**4**	40.3	-	40.3	-	40.0	-
**5**	57.3	0.77 (m)	57.2	0.77 (m)	57.1	0.78 (m)
**6**	19.4	1.61 (m)	19.3	1.59 (m)	19.4	1.58 (m)
**7**	34.0	1.40 (m),1.55 (m)	34.0	1.30 (m), 1.53 (m)	34.0	1.38 (m), 1.55 (m)
**8**	40.6	-	40.6	-	40.6	-
**9**	48.5	1.61 (m)	48.5	1.62 (m)	48.5	1.61 (m)
**10**	37.9	-	37.9	-	37.9	-
**11**	24.5	1.88 (m),1.90 (m)	24.5	1.88 (m), 1.93 (m)	24.5	1.84 (m), 1.90 (m)
**12**	123.6	5.24 br. s	123.6	5.24 br. s	123.6	5.24 br. s
**13**	145.2	-	145.1	-	145.2	-
**14**	42.8	-	42.7	-	42.7	-
**15**	28.8	1.80 (m),2.01 (m)	28.8	1.81 (m)	28.8	1.75 (m)
**16**	24.0	1.80 (m),1.90 (m)	24.0	1.82 (m), 1.94 (m)	24.0	1.83 (m), 2.01 (m)
**17**	47.7	-	47.6	-	47.6	-
**18**	42.9	2.85 (d, 10.5)	42.9	2.84 (d, 10.5)	42.9	2.84 (d, 11.0)
**19**	47.3	1.19 (m),1.72 (m)	47.2	1.17 (m), 1.73 (m)	47.2	1.08 (m), 1.76 (m)
**20**	31.6	-	31.6	-	31.6	-
**21**	34.9	0.98 (m),1.29 (m)	34.9	0.98 (m), 1.29 (m)	34.9	1.12 (m), 1.43 (m)
**22**	33.8	1.49 (m),1.81 (m)	33.8	1.50 (m), 1.84 (m)	33.8	1.50 (m), 1.89 (m)
**23**	28.6	1.06 (s)	28.5	1.06 (s)	28.7	1.04 (s)
**24**	17.2	0.86 (s)	17.2	0.86 (s)	17.2	0.86 (s)
**25**	16.0	0.94 (s)	16.0	0.94 (s)	16.0	0.93 (s)
**26**	17.7	0.81 (s)	17.7	0.81 (s)	17.7	0.81 (s)
**27**	26.4	1.16 (s)	26.4	1.17 (s)	26.4	1.17 (s)
**28**	181.9	-	181.8	-	181.8	-
**29**	33.6	0.94 (s)	33.6	0.94 (s)	33.6	0.95 (s)
**30**	24.1	0.91 (s)	24.1	0.91 (s)	24.1	0.91 (s)
	3-Xyl		3-Xyl		3-Ara	
**1**	106.3	4.38 (d, 7.0)	106.5	4.38 (d, 7.0)	105.2	4.51 (d, 5.0)
**2**	78.9	3.44 (m)	78.8	3.46 (m)	76.5	3.76 (m)
**3**	78.4	3.35 (m)	78.5	3.33 (m)	72.5	3.70 (m)
**4**	72.5	3.41 (m)	72.6	3.41 (m)	68.5	3.98 (t)
**5**	66.5	3.85 (m)	66.6	3.86 (m)	64.5	3.88(m), 3.52 (m)
	Rha		Rha		Rha	
**1**	101.5	5.36 (s)	101.6	5.30 (s)	101.7	5.17 (s)
**2**	71.6	4.09 br. s	71.0	4.27 br. s	71.9	4.04 br. s
**3**	80.8	3.86 (m)	82.9	3.88 (m)	80.7	3.81 (m)
**4**	73.0	3.53 (m)	72.7	4.08 (m)	73.0	3.52 (m)

**5**	70.1	3.87 (m)	70.0	3.96 (m)	70.3	3.88 (m)
**6**	18.0	1.23 (d, 6.0)	18.2	1.23 (d, 10.5)	18.0	1.23 (d, 6.0)
	Rib		Glc		Rib	
**1**	104.4	4.99 (d, 4.0)	103.3	4.84 (d, 8.0)	104.2	5.00 (d, 3.5)
**2**	71.7	3.68 (m)	71.6	3.17 (m)	73.7	3.70 (m)
**3**	68.7	3.76 (m)	68.4	3.56 (m)	69.0	3.75 (m)
**4**	70.2	3.88 (m)	75.3	3.67 (m)	70.2	3.90 (m)
**5**	65.1	3.68 (m),3.88 (m)	79.5	3.72 (m)	65.1	3.70(m), 3.91 (m)
**6**			62.7	3.84(m), 3.67(m)		

measured in methanol-d_4

Table S2. ^{1}H **and** ^{13}C **NMR (500/125 MHz) data of 4-6,** δ **in ppm,** J **in Hz**

**NO.**	**4**		**5**		**6**	
	δ_C	δ_H	δ_C	δ_H	δ_C	δ_H
**1**	39.4	1.50 (m)	39.4	0.98 (m)	39.3	1.00 (m), 1.57 (m)
**2**	27.4	1.65 (m),1.81 (m)	27.2	1.91 (m)	27.2	1.67 (m), 1.76 (m)
**3**	89.1	3.33 (dd, 4.0, 11.5)	89.2	3.31 (dd, 4.0, 11.5)	89.1	3.37 (dd, 3.5, 11.5)
**4**	40.1	-	40.1	-	40.1	-
**5**	56.5	0.80 (m)	56.5	0.80 (d, 12.0)	56.4	0.84 (d, 12.0)
**6**	19.1	1.23 (m),1.50 (m)	19.0	1.22 (m), 1.46 (m)	19.0	1.19 (m), 1.48 (m)
**7**	33.7	1.32 (m), 1.52 (m)	33.6	1.30 (m), 1.46 (m)	33.6	1.35 (m), 1.51 (m)
**8**	40.2	-	40.4	-	40.4	-
**9**	48.5	1.64 (d, 6.4)	48.6	1.64 (m)	48.6	1.67 (m)
**10**	37.5	-	37.5	-	37.5	-
**11**	24.3	1.84 (m), 1.91 (m)	24.3	1.83 (m), 1.91 (m)	23.9	1.79 (m), 1.93 (m)
**12**	123.0	5.46 br. s	123.4	5.44 br. s	123.4	5.43 br. s
**13**	145.3	-	144.6	-	144.6	-
**14**	42.7	-	42.6	-	42.6	-
**15**	28.8	1.21 (m), 2.14 (m)	28.7	1.17 (m),2.36 (m)	28.8	1.16 (m), 2.33 (m)
**16**	24.2	1.98 (m), 2.04 (m)	23.9	1.97 (m),2.08 (m)	24.2	1.93 (m), 2.09 (m)
**17**	47.2	-	47.5	-	47.5	-
**18**	42.5	3.28 (dd, 3.5, 13.0)	42.2	3.21 (dd, 3.5, 13.5)	42.2	3.21 (dd, 7.0, 13.0)
**19**	47.0	1.21 (m), 1.76 (m)	46.7	1.25 (m), 1.76 (m)	46.7	1.25 (m), 1.76 (m)
**20**	31.5	-	31.3	-	31.2	-
**21**	34.7	1.46 (m)	34.5	0.98 (m), 1.40 (m)	34.5	1.00 (m), 1.42 (m)
**22**	33.7	1.78 (m), 1.83 (m)	33.0	1.78 (m), 1.83 (m)	33.0	1.76 (m), 1.88 (m)
**23**	28.8	1.38 (s)	28.7	1.33 (s)	28.7	1.32 (s)
**24**	17.7	1.18 (s)	17.7	1.17 (s)	17.5	1.12 (s)
**25**	16.1	0.83 (s)	16.1	0.89 (s)	16.1	0.91 (s)
**26**	17.9	1.01 (s)	18.0	1.11 (s)	18.0	1.02 (s)
**27**	26.7	1.30 (s)	26.6	1.29 (s)	26.5	1.28 (s)
**28**	180.7	-	176.9	-	177.0	-
**29**	33.8	0.98 (s)	33.6	0.93 (s)	33.6	0.92 (s)
**30**	24.2	0.96 (s)	24.1	0.90 (s)	24.3	0.92 (s)

	3-Xyl		3-Ara		3-Xyl	
**1**	106.7	4.81 (d, 6.5)	105.8	4.86 (d, 5.0)	108.1	4.81 (d, 7.5)
**2**	77.9	4.27 (m)	75.8	4.54 (m)	78.8	4.24 (m)
**3**	79.9	4.16 (m)	75.3	4.25 (m)	74.5	4.18 (m)
**4**	72.1	4.1 4(m)	69.4	4.33 (m)	71.7	4.14 (m)
**5**	67.5	3.69 (t), 4.33 (m)	66.3	3.82 (m)	67.6	3.79 (t), 4.34 (m)
	Rha		Rha		28-Glc	
**1**	102.0	6.47 (s)	101.9	6.68 (s)	96.1	6.25 (d, 8.0)
**2**	72.0	5.09 br. s	72.6	4.94 br. s	74.4	4.12 (m)
**3**	83.6	4.80 (d, 6.5)	81.8	4.65 (m)	79.2	4.22 (m)
**4**	73.3	4.33 (m)	73.4	4.46 (m)	71.4	4.41 (m)
**5**	70.3	4.72 (m)	70.4	4.78 (m)	78.5	4.09 (m)
**6**	19.0	1.44 (d, 6.0)	18.9	1.56 (d, 6.0)	69.7	4.46 (m), 4.34 (m)
	Gal		Rib		Glc	
**1**	104.8	5.90 (d, 8.0)	105.2	5.99 (d, 4.5)	105.1	5.00 (d, 8.0)
**2**	73.5	4.05 (m)	73.3	4.31 (m)	75.8	3.95 (t)
**3**	73.3	4.70 (m)	69.9	4.50 (m)	77.0	4.14 (m)
**4**	69.2	4.28 (m)	70.8	4.19 (m),4.33 (m)	79.1	4.40 (m)
**5**	76.5	4.49 (m)	65.8	4.17 (m)	77.7	3.68 (m)
**6**	63.1	4.40 (m)	-		61.8	4.03 (t), 4.18 (m)
			28-Glc		Rha	
**1**			96.3	6.36 (d, 8.5)	103.2	5.86 br. s
**2**			74.6	4.30 (m)	73.2	4.68 (m)
**3**			79.4	4.20 (m)	73.3	4.55 (m)
**4**			71.6	4.38 (m)	74.5	4.34 (m)
**5**			79.8	4.06 (m)	70.8	4.97 (m)
**6**			62.7	4.33 (m), 4.46 (m)	19.0	1.71 (d, 6.0)

measured in pyridine-d_5

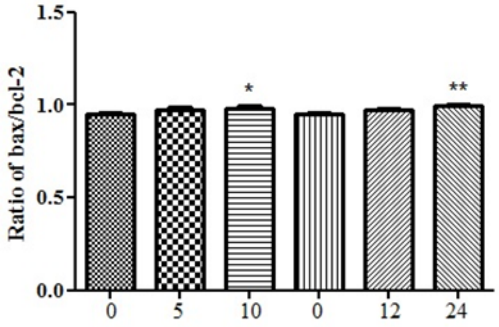

Figure S1 Analysis of the ratio of bax/bcl-2 in compounds **1** and **2** treatment groups. *P < 0.05, **P < 0.01.

Thanks again for all your excellent comments and suggestions

Compound 1

HPLC was performed on a Shimadzu LC-20A pump system (Shimadzu Corporation, Tokyo, Japan), equipped with an SPD-M20A photodiode array detector monitoring, analytical RP-HPLC column (Agilent XDB-C_{18}, 250 × 4.6 mm, 5 μm).

50% ACN-H_2O 210 nm t_R= 12.435min 1 ml/min

Compound 1

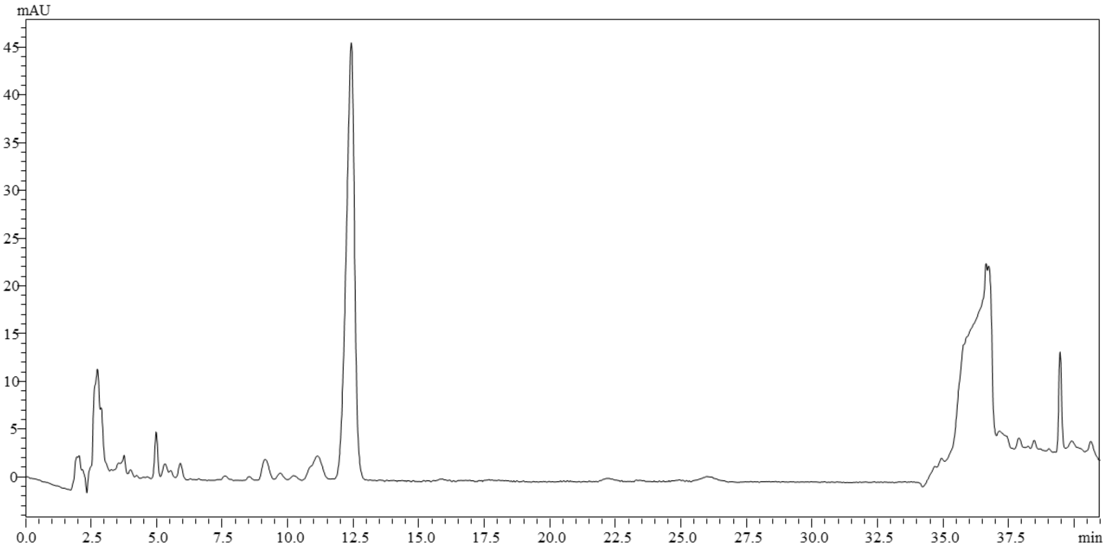

Figure S2 HPLC-PDA (210 nm) profiles of **1**

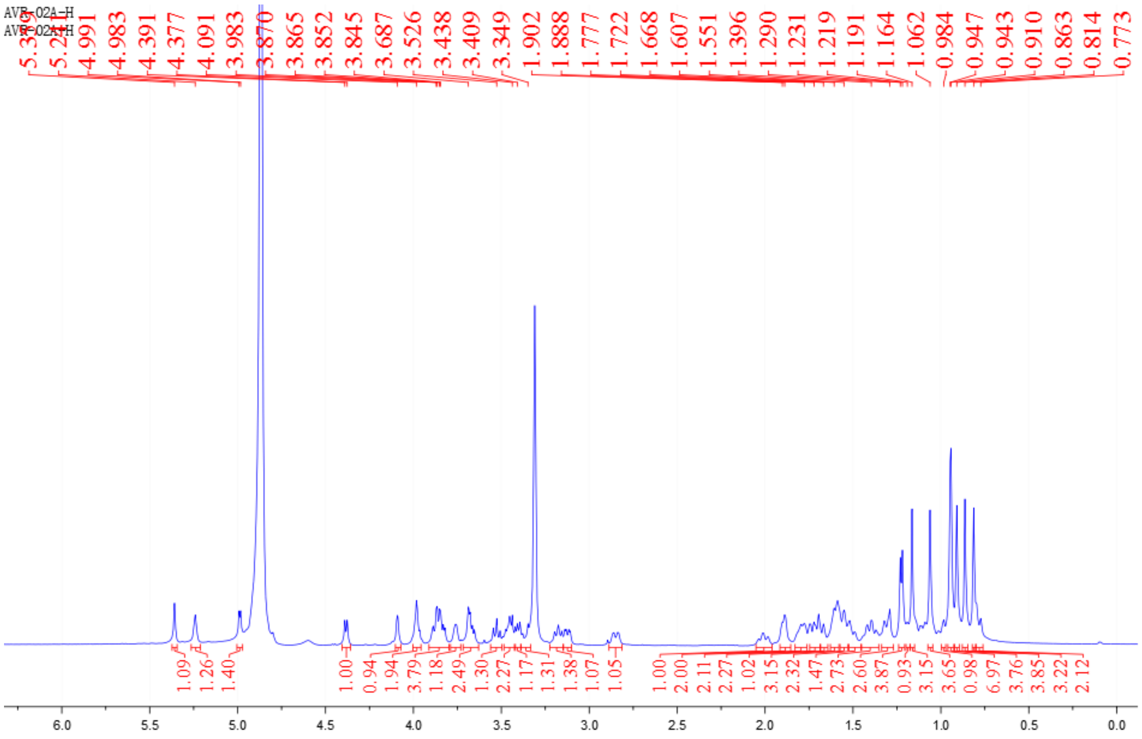

Figure S3 ^{1}H NMR spectrum of **1** in CD_3OD (500 MHz)

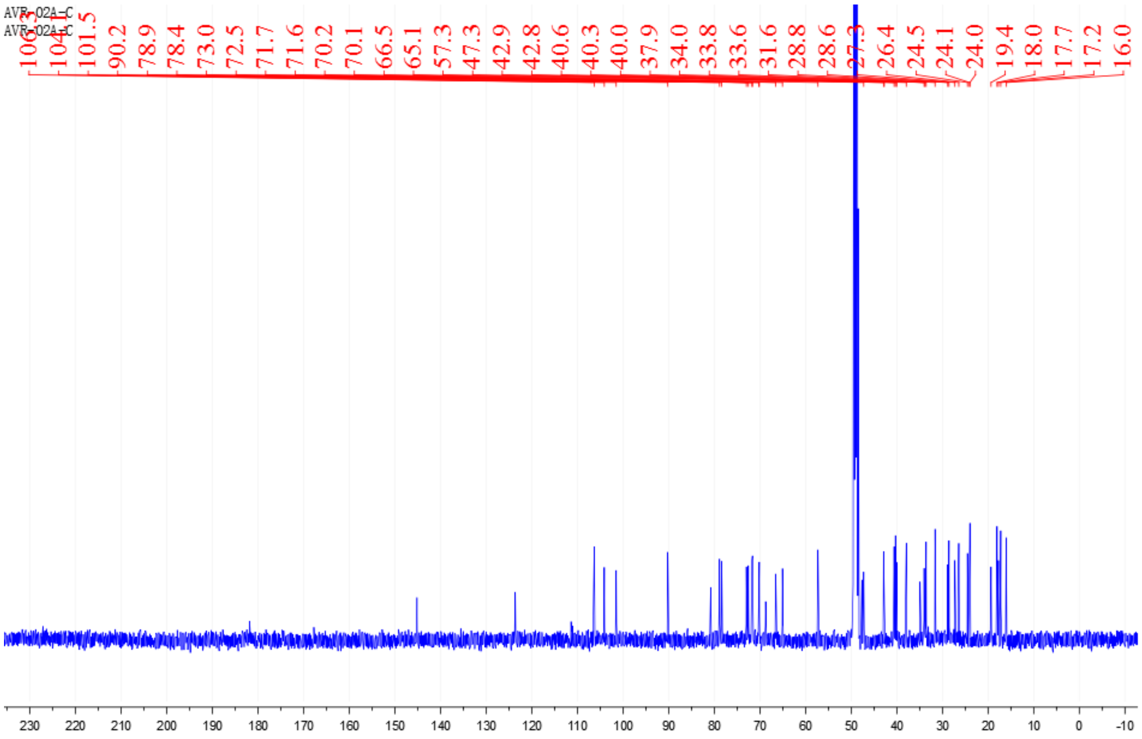

Figure S4 ^{13}C NMR spectrum of **1** in CD_3OD (125 MHz)

Compound 2

50% ACN-H_2O 210 nm t_R=12.714 min   1 ml/min

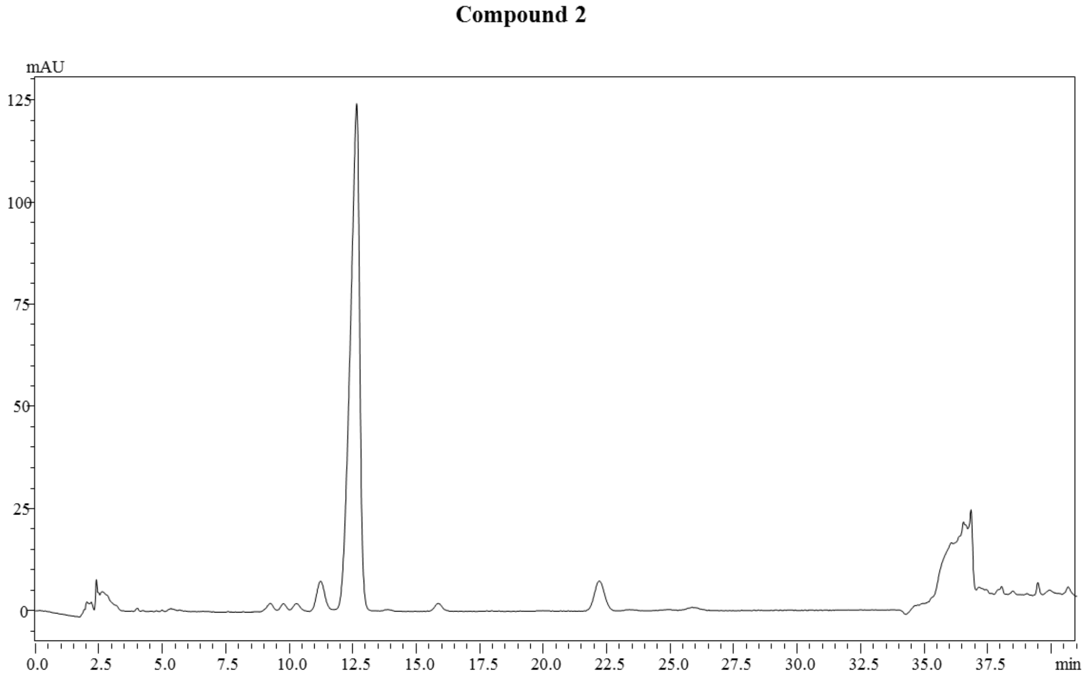

Figure S5 HPLC-PDA (210 nm) profiles of **2**

Formula Predictor Report - AVR-02_3.lcd

Page 1 of 1

Data File: D:\Datas\叶云云\新建文件夹\AVR-02_3.lcd

Elmt	Val.	Min	Max	Elmt	Val.	Min	Max	Elmt	Val.	Min	Max	Use Adduct
H	1	22	100	F	1	0	0	Br	1	0	0	H
C	4	17	60	P	3	0	0					HCOO
N	3	0	0	S	2	0	0					Cl
O	2	0	30	Cl	1	0	0					CF3COO

Error Margin (ppm):	100	DBE Range:	-2.0 - 1200.0	Electron Ions:	both
HC Ratio:	unlimited	Apply N Rule:	no	Use MSn Info:	no
Max Isotopes:	all	Isotope RI (%):	1.00	Isotope Res:	10000
MSn Iso RI (%):	75.00	MSn Logic Mode:	AND	Max Results:	100

Event#: 3 MS(E-) Ret. Time : 11.218 Scan# : 1998

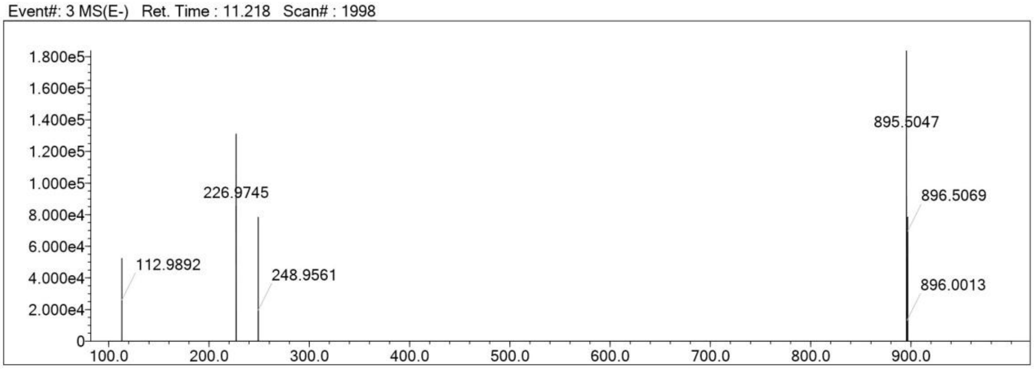

Measured region for 895.5047 m/z

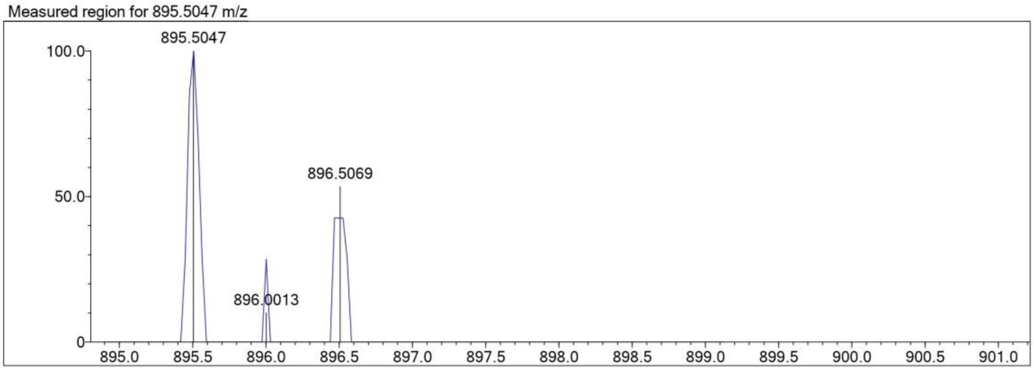

C47 H76 O16 [M-H]- : Predicted region for 895.5061 m/z

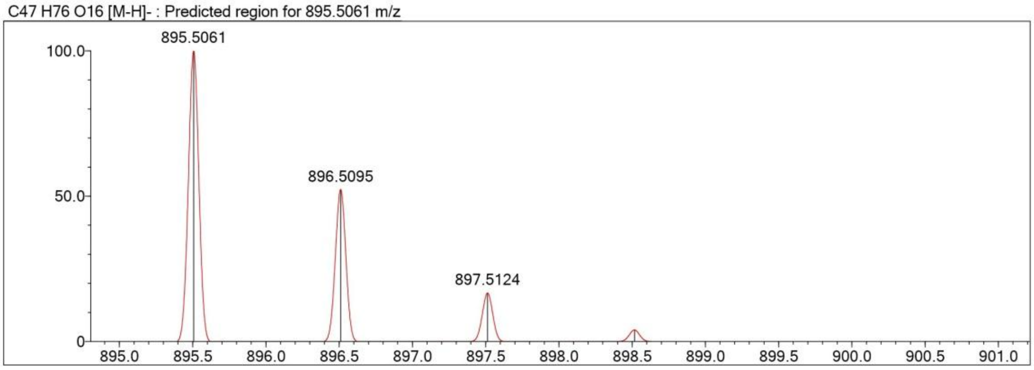

Rank	Score	Formula (M)	Ion	Meas. m/z	Pred. m/z	Df. (mDa)	Df. (ppm)	Iso	DBE
2	0.00	C47 H76 O16	[M-H]-	895.5047	895.5061	-1.4	-1.56	0.00	10.0

Figure S6 HR-ESI-MS spectrum of **2**

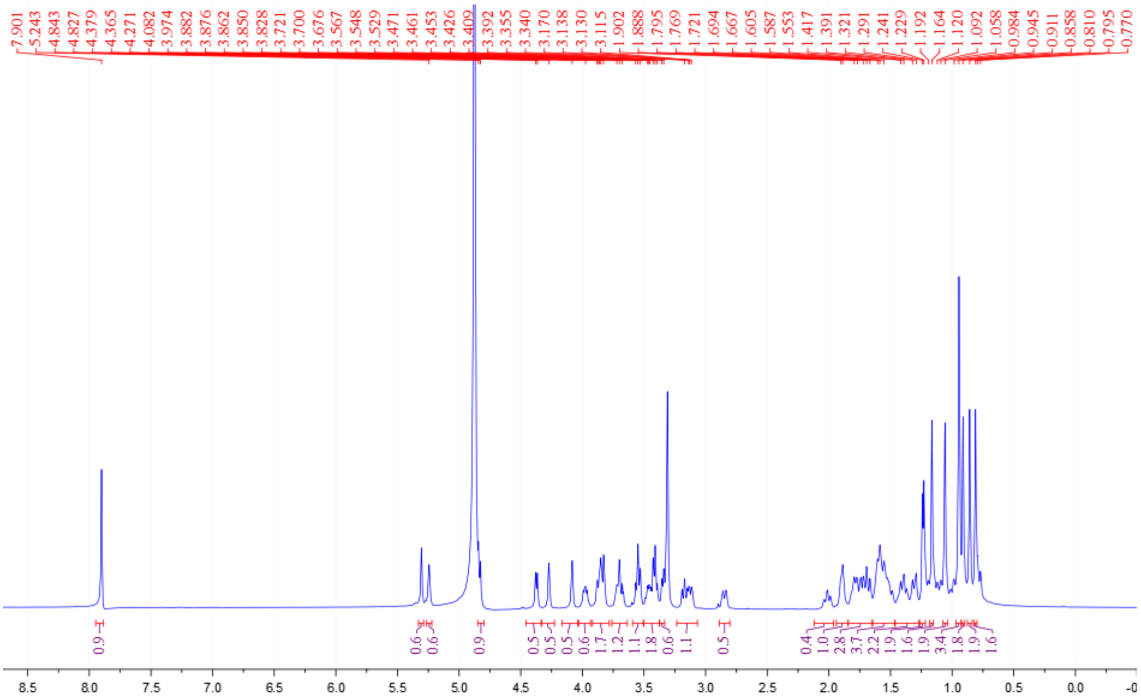

Figure S7 ^{1}H NMR spectrum of **2** in CD_3OD (500 MHz)

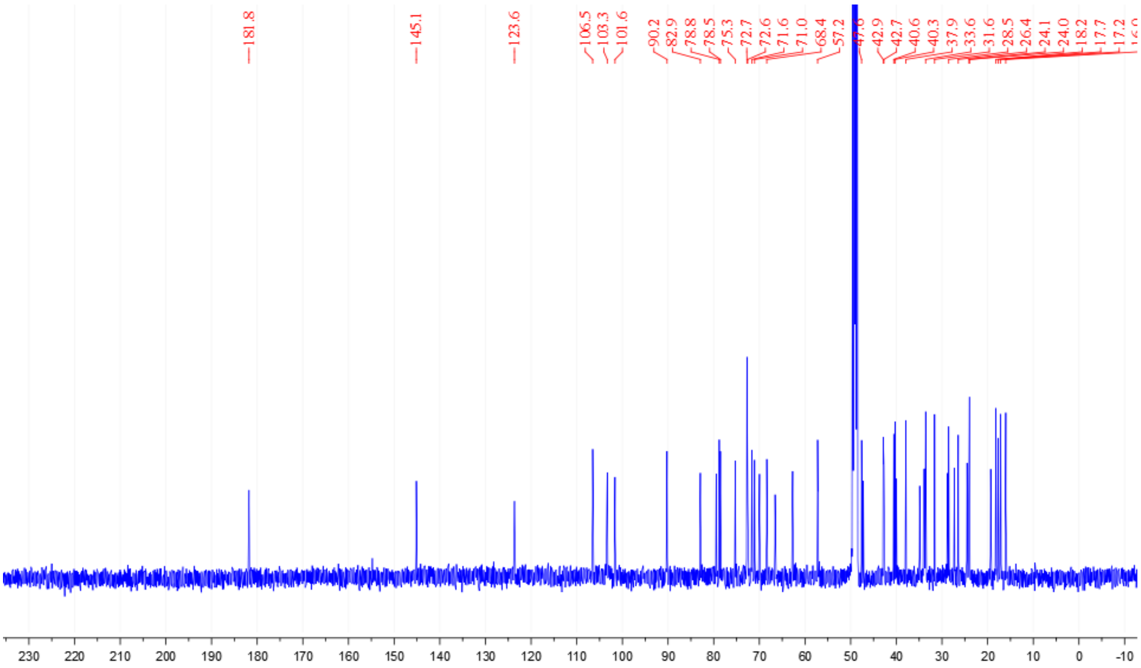

Figure S8 ^{13}C NMR spectrum of **2** in CD_3OD (125 MHz)

Data File: D:\Datas\叶云云\新建文件夹\AVR-01_2.lcd

Elmt	Val.	Min	Max	Elmt	Val.	Min	Max	Elmt	Val.	Min	Max	Use Adduct
H	1	22	100	F	1	0	0	Br	1	0	0	H
C	4	17	60	P	3	0	0					HCOO
N	3	0	0	S	2	0	0					Cl
O	2	0	30	Cl	1	0	0					CF3COO

Error Margin (ppm):	100	DBE Range:	-2.0 - 1200.0	Electron Ions:	both
HC Ratio:	unlimited	Apply N Rule:	no	Use MSn Info:	no
Max Isotopes:	all	Isotope RI (%):	1.00	Isotope Res:	10000
MSn Iso RI (%):	75.00	MSn Logic Mode:	AND	Max Results:	100

Event#: 3 MS(E-) Ret. Time : 11.508 Scan# : 2058

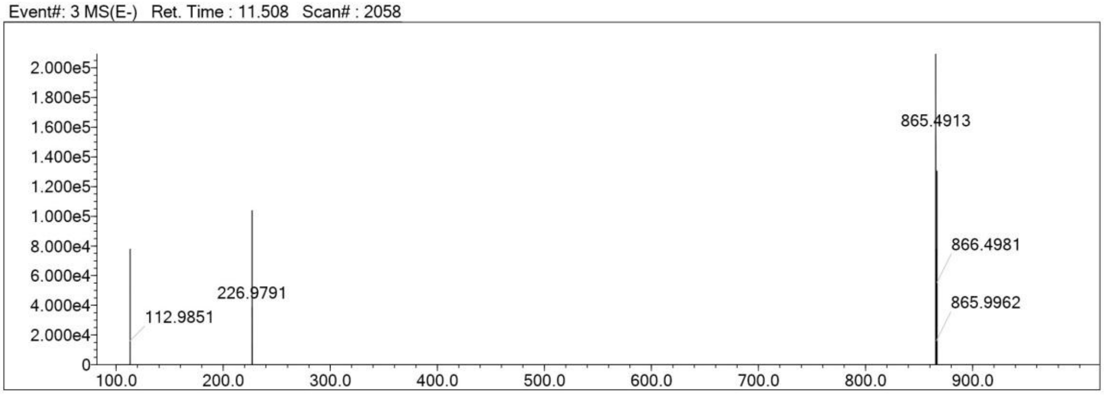

Measured region for 865.4913 m/z

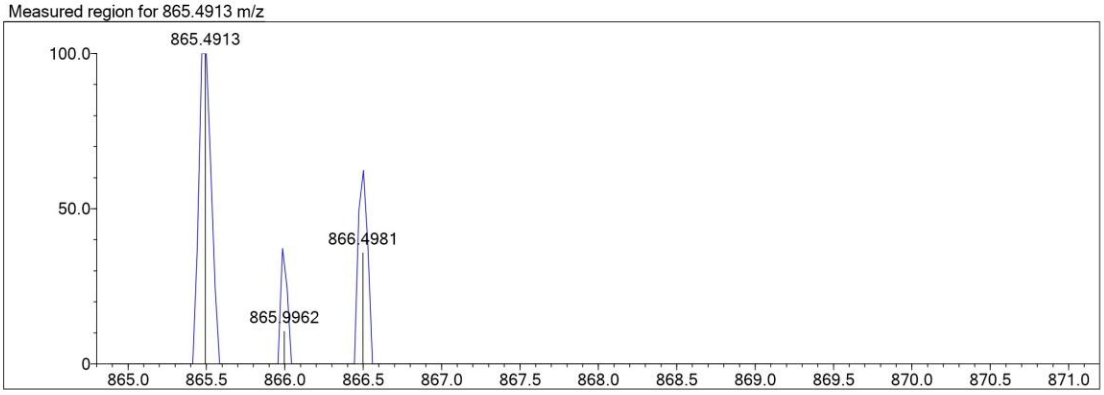

C46 H74 O15 [M-H]- : Predicted region for 865.4955 m/z

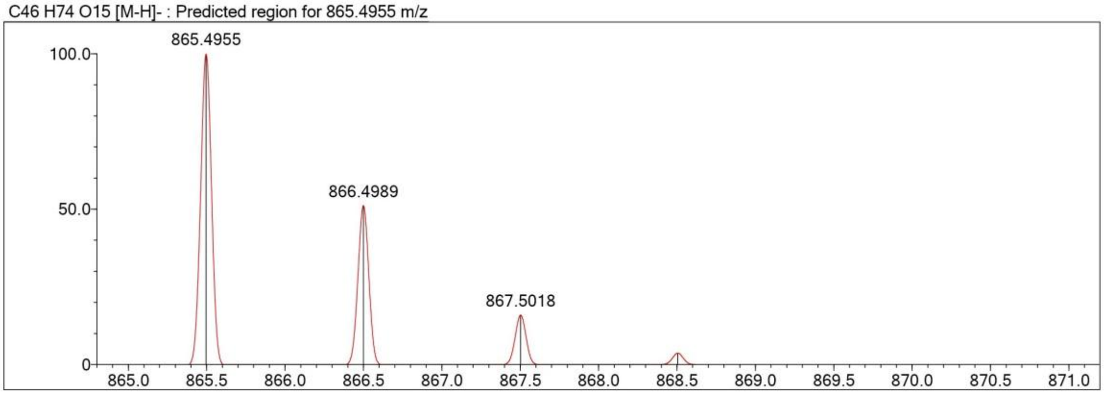

Rank	Score	Formula (M)	Ion	Meas. m/z	Pred. m/z	Df. (mDa)	Df. (ppm)	Iso	DBE
5	0.00	C46 H74 O15	[M-H]-	865.4913	865.4955	-4.2	-4.85	0.00	10.0

Figure S9 HR-ESI-MS spectrum of **3**

Compound 3

50% ACN-H_2O 210 nm t_R= 12.260min 1 ml/min

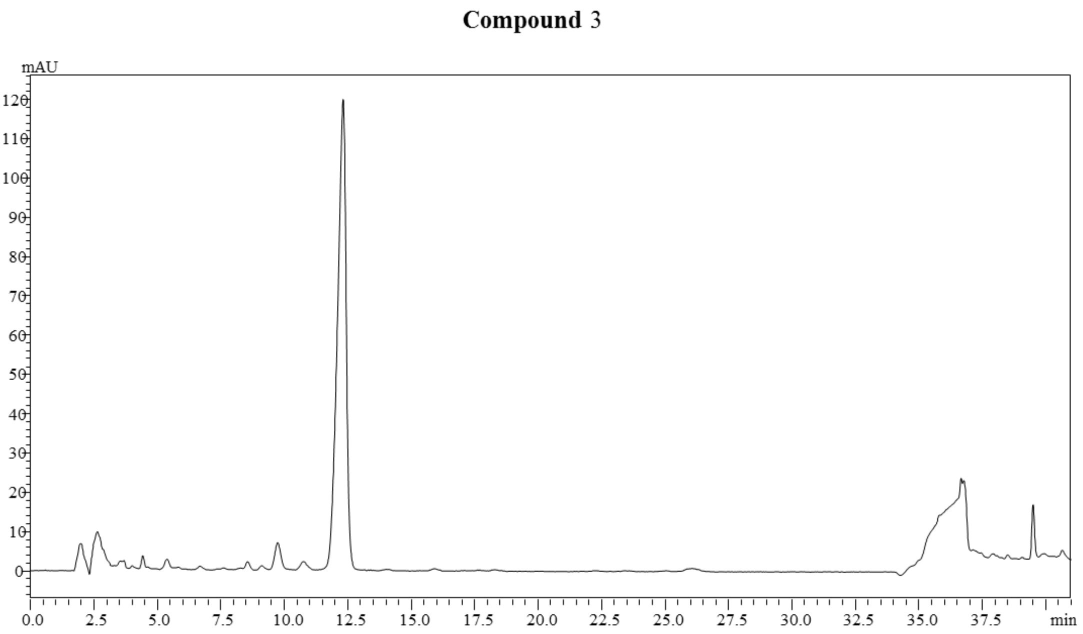

Figure S10 HPLC-PDA (210 nm) profiles of **3**

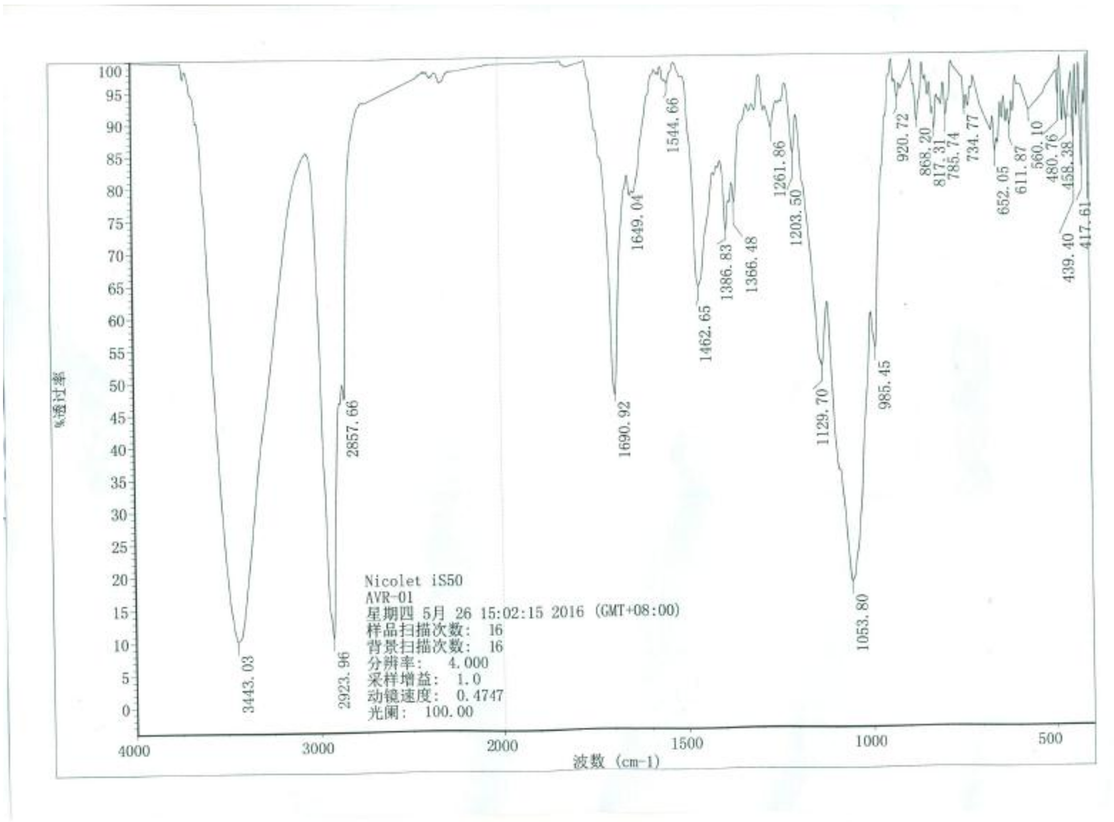

Figure S11 IR spectrum of **3**

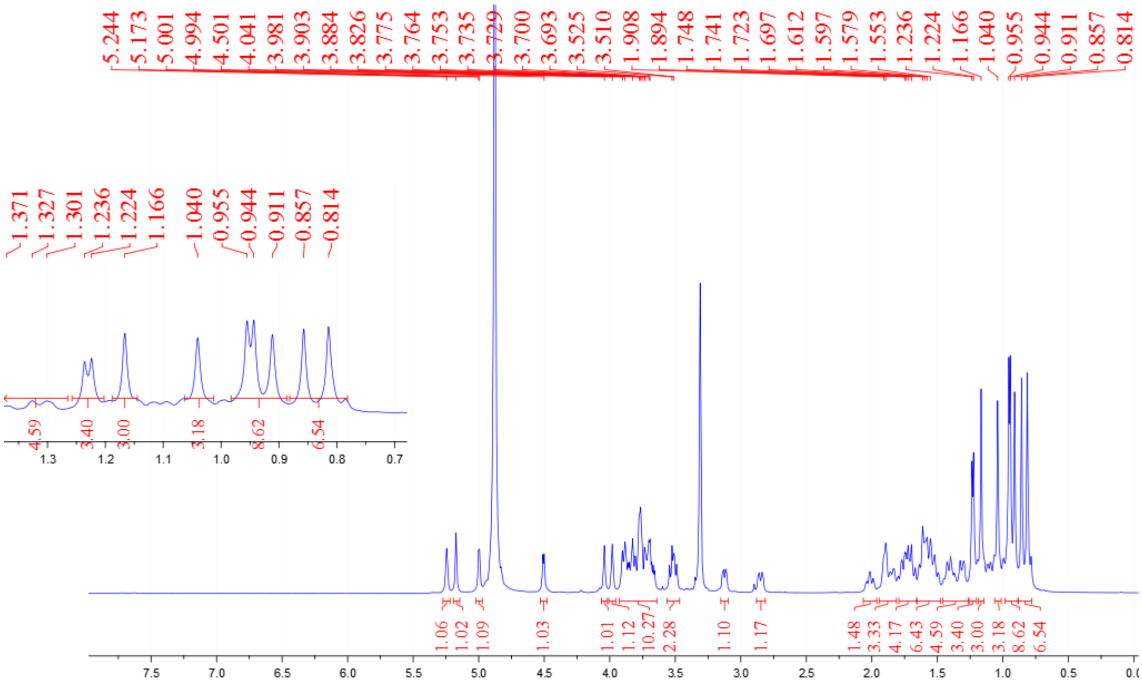

Figure S12 ^{1}H NMR spectrum of **3** in CD_3OD (500 MHz)

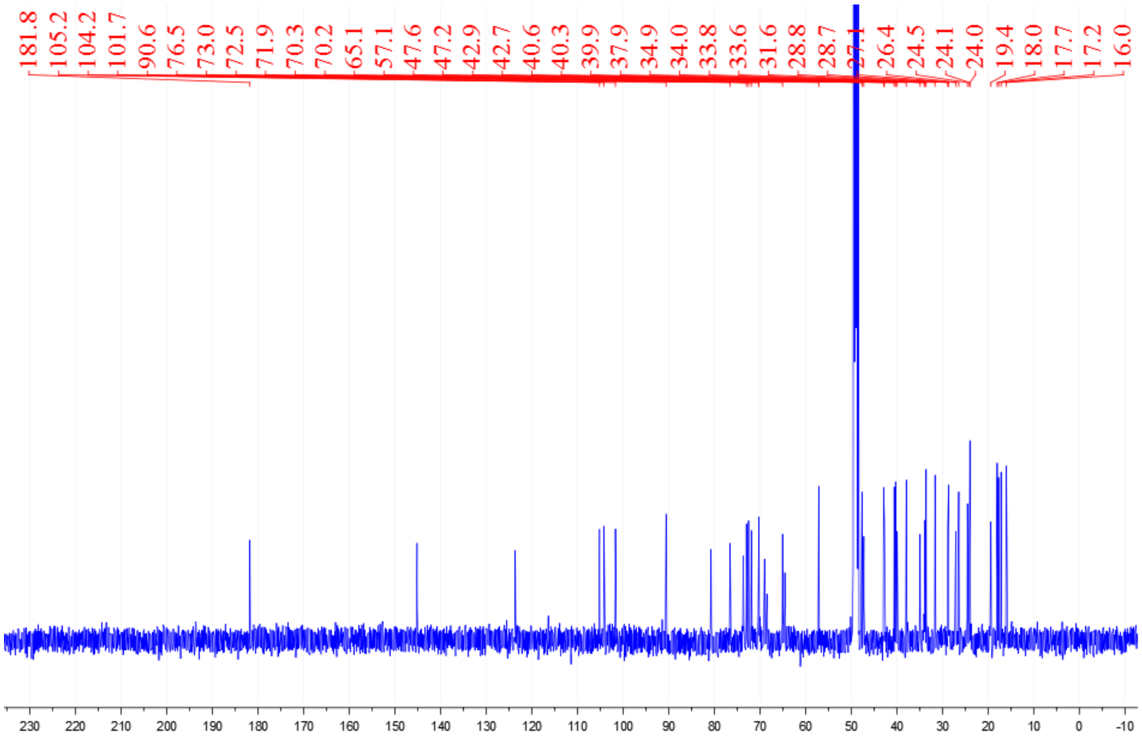

Figure S13 ^{13}C NMR spectrum of **3** in CD_3OD (125 MHz)

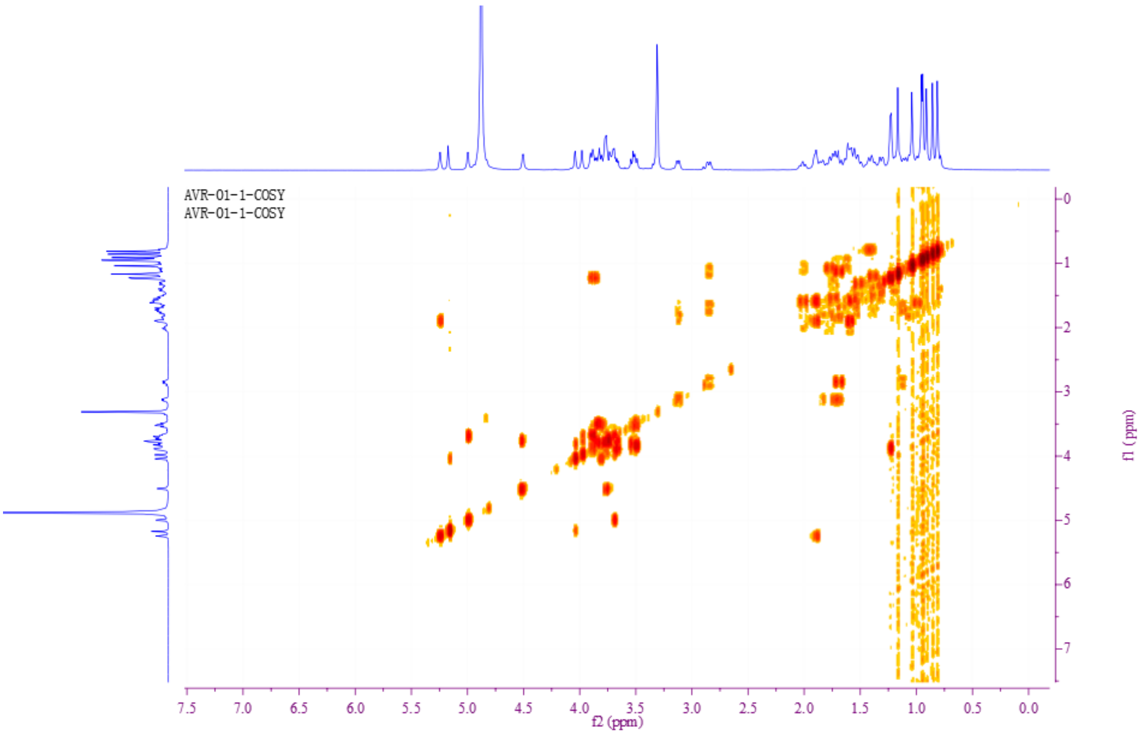

Figure S14 ^{1}H-^{1}H COSY spectrum of **3**

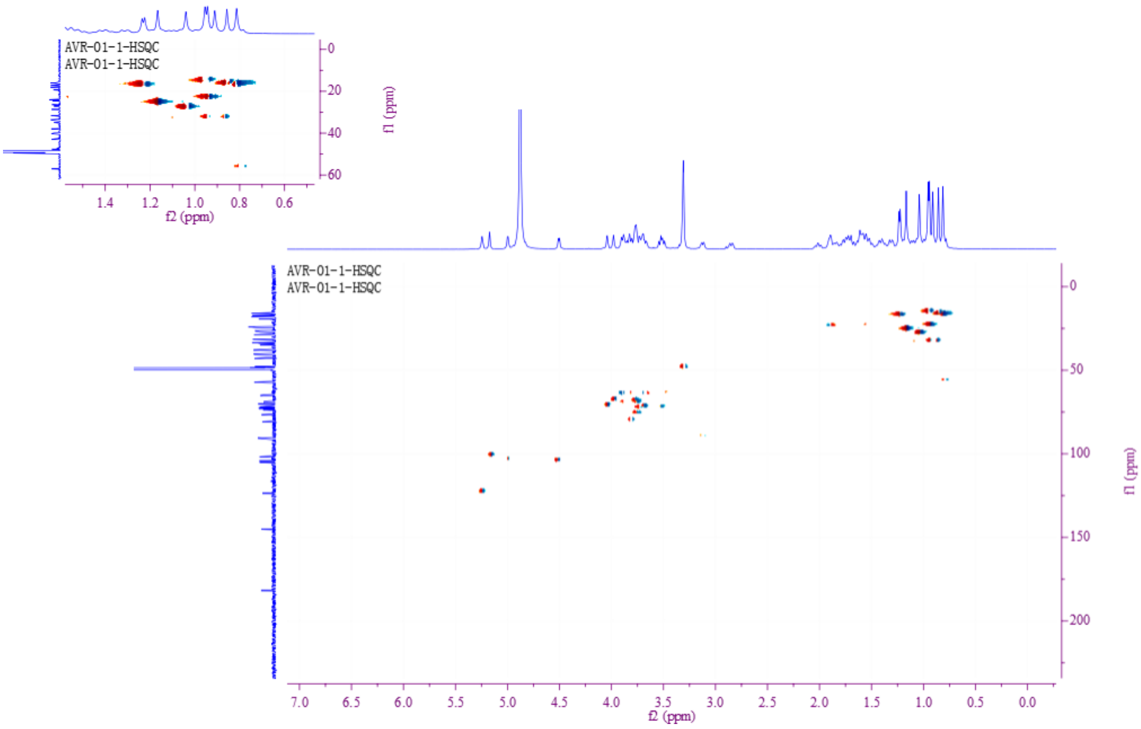

Figure S15 HSQC spectrum of **3**

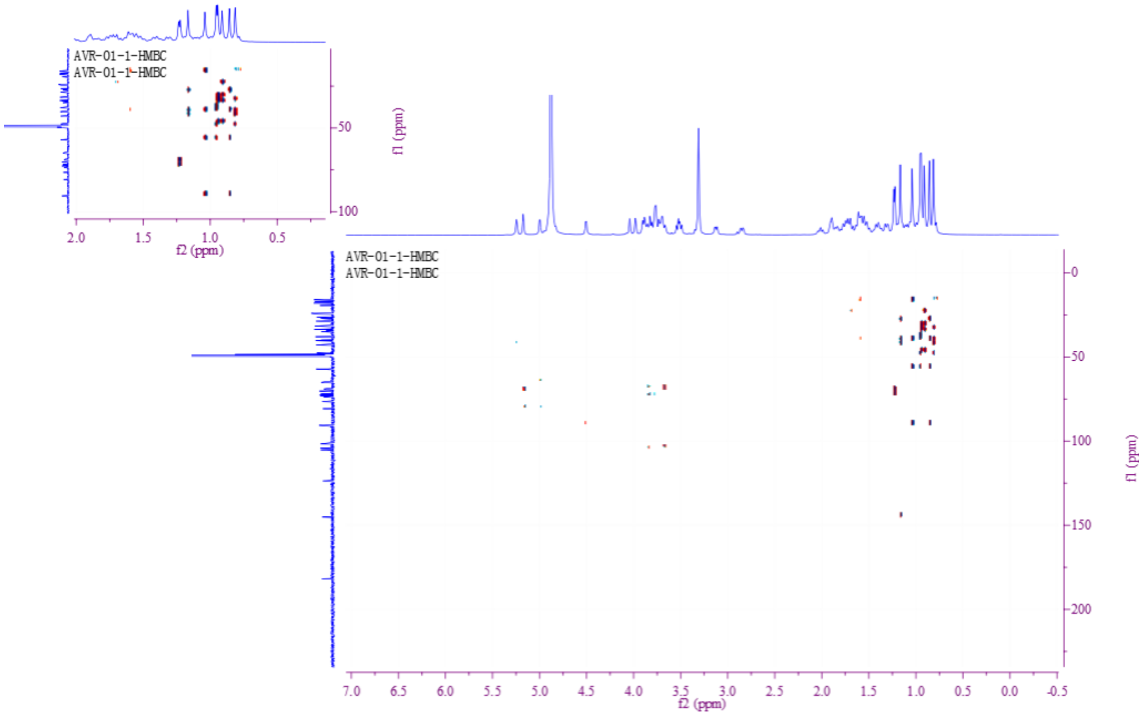

Figure S16 HMBC spectrum of **3**

Determination of Sugar Configuration:

Sugar was dissolved in pyridine (1.0 ml) containing L-cysteine methyl ester hydrochloride (5.0 mg) and heated at 60 ℃ for 1 h. A 0.05 ml solution of o-torylisothiocyanate (5.0 mg) in pyridine was added to the mixture, which was heated at 60 ℃ for 1 h. The reaction mixture was directly analyzed by reversed-phase HPLC. HPLC was performed on a Shimadzu LC-20A pump system (Shimadzu Corporation, Tokyo, Japan), equipped with an SPD-M20A photodiode array detector monitoring, analytical RP-HPLC column (Agilent XDB-C_{18}, 250 × 4.6 mm, 5 μm). 25% CH_3CN for 35 min and subsequent washing of the column with 95% CH_3CN at a flow rate 0.8 ml/min.

The glycoside (2.0 mg) were hydrolyzed in 2 M HCl (10.0 ml) and heated at 80 ℃ for 4h, then concentrated to dryness. The residue was dissolved in pyridine (1.0 ml) containing L-cysteine methyl ester hydrochloride (5.0 mg) and heated at 60 ℃ for 1 h. A 0.05 ml solution of o-torylisothiocyanate (5.0 mg) in pyridine was added to the mixture, which was heated at 60 ℃ for 1 h. The reaction mixture was directly analyzed by reversed-phase HPLC. HPLC was performed on a Shimadzu LC-20A pump system (Shimadzu Corporation, Tokyo, Japan), equipped with

an SPD-M20A photodiode array detector monitoring, analytical RP-HPLC column (Agilent XDB-C_{18}, 250 × 4.6 mm, 5 μm). 25% CH_3CN for 35 min and subsequent washing of the column with 95% CH_3CN at a flow rate 0.8 ml/min.

Compared with the standard sugar and glycoside retention time, identified the type and number of sugar.

25% ACN-H_2O 0.8 ml/min 254 nm

Compound 3

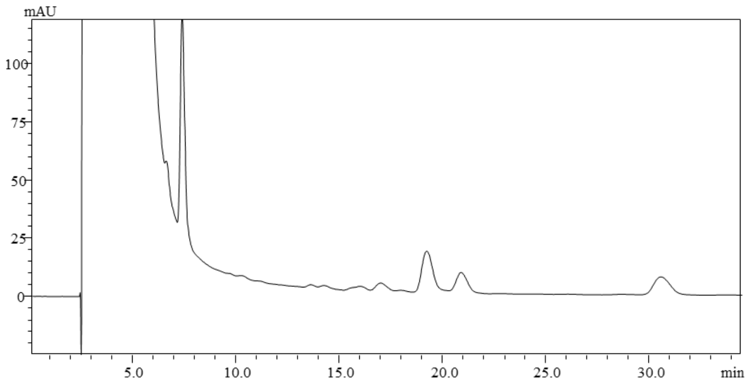

分析: t_{R1}=19.241 min (α-L-Ara)

t_{R2}=20.906 min (β-D-rib)

t_{R3}=30.504 min (α-L-Rha)

Figure S17 general acid hydrolysis of **3**

Compound 4

50% ACN-H_2O 210 nm t_R=12.518 min 1 ml/min

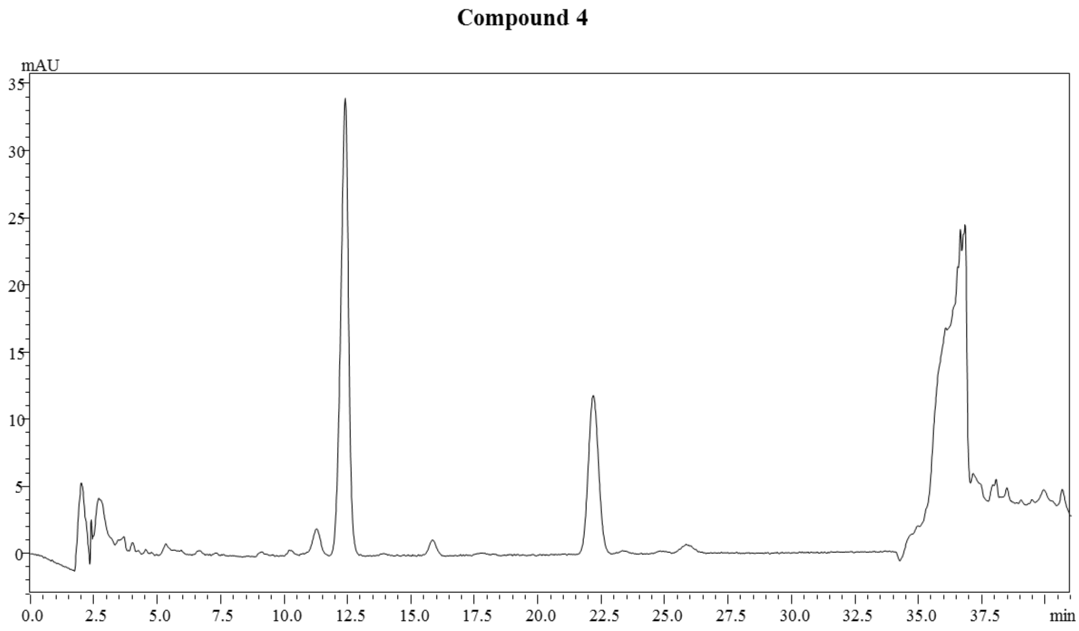

Figure S18 HPLC-PDA (210 nm) profiles of **4**

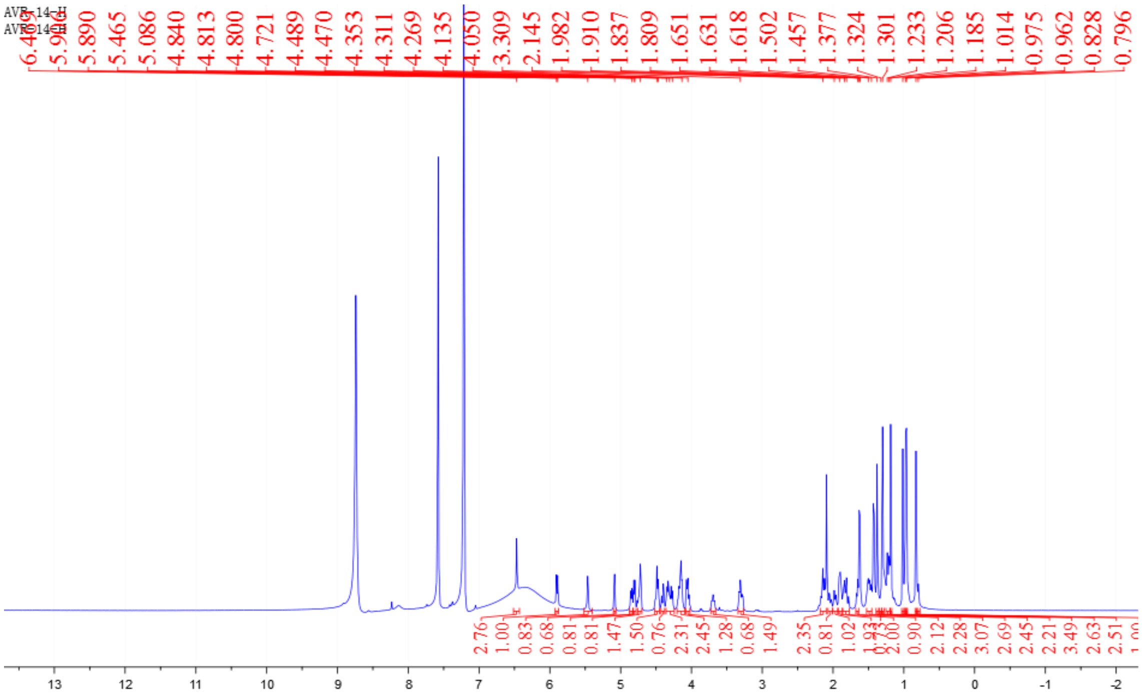

Figure S19 ^{1}H NMR spectrum of **4** in Pyridine-d_5 (500 MHz)

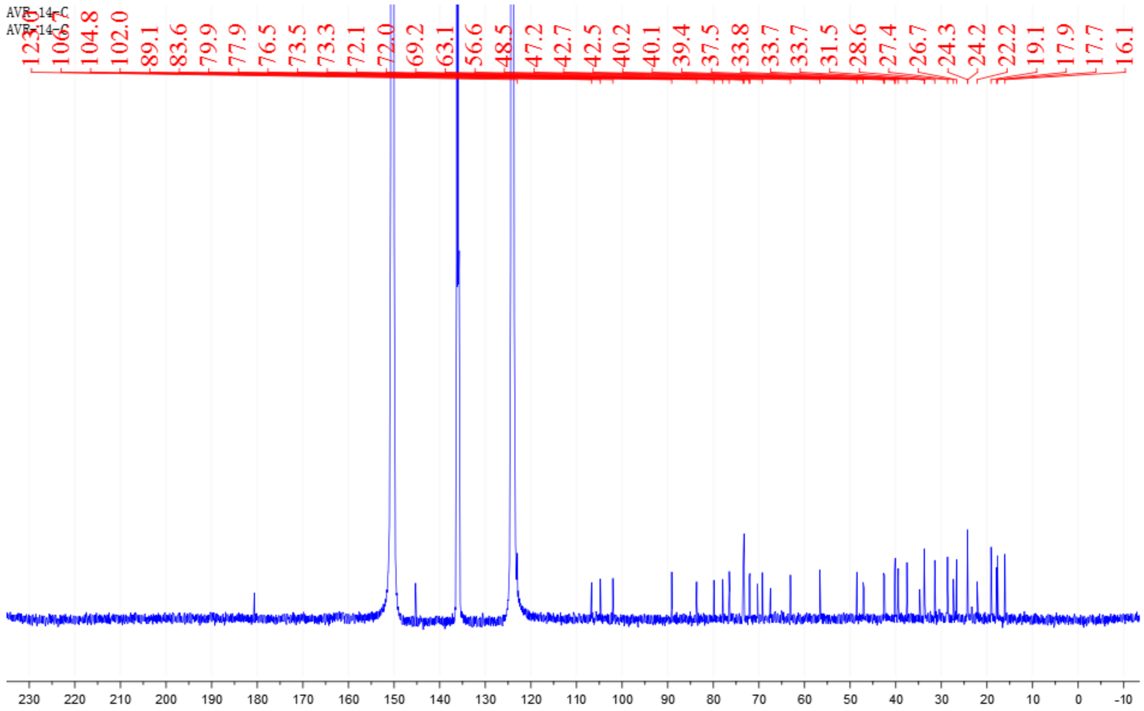

Figure S20 ^{13}C NMR spectrum of **4** in Pyridine-d_5 (125 MHz)

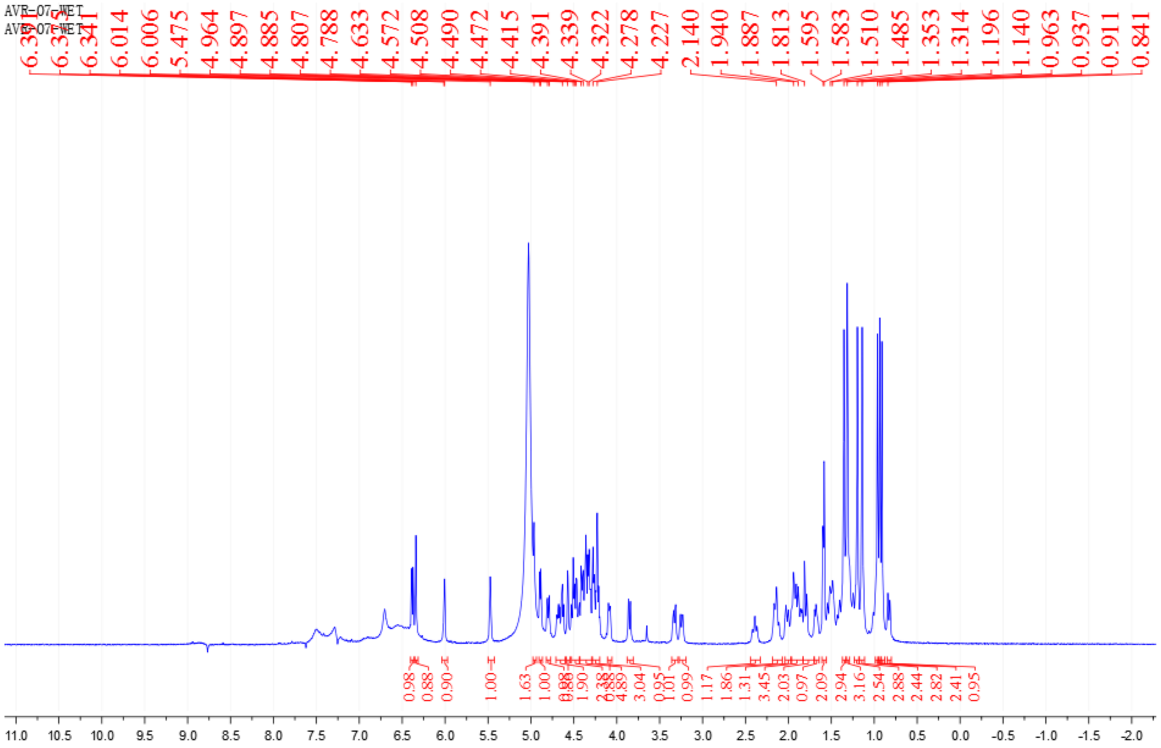

Figure S21 ^{1}H NMR spectrum of **5** in Pyridine-d_5 (500 MHz)

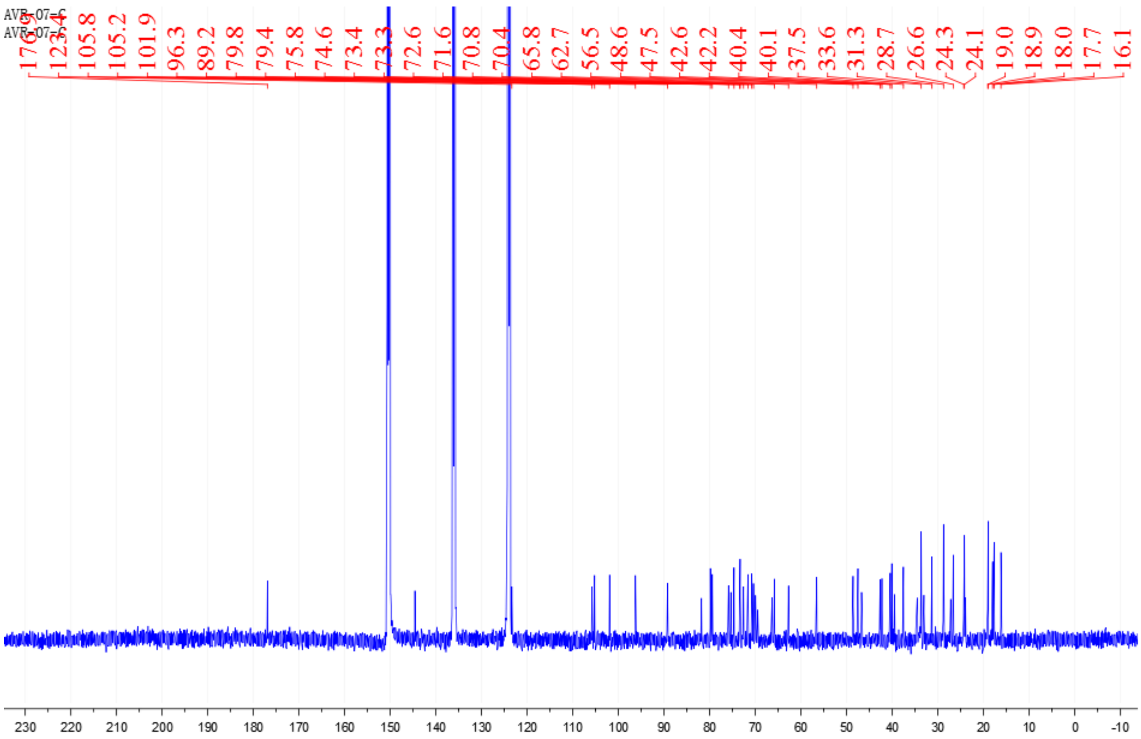

Figure S22 ^{13}C NMR spectrum of **5** in Pyridine-d_5 (125 MHz)

Data File: D:\Datas\叶云云\ic-3_1.lcd

Elmt	Val.	Min	Max	Elmt	Val.	Min	Max	Elmt	Val.	Min	Max	Use Adduct
H	1	22	100	F	1	0	0	Br	1	0	0	H
C	4	17	60	P	3	0	0					HCOO
N	3	0	0	S	2	0	0					Cl
O	2	0	30	Cl	1	0	0					CF3COO

Error Margin (ppm):	100	DBE Range:	-2.0 - 1200.0	Electron Ions:	both
HC Ratio:	unlimited	Apply N Rule:	no	Use MSn Info:	no
Max Isotopes:	all	Isotope RI (%):	1.00	Isotope Res:	10000
MSn Iso RI (%):	75.00	MSn Logic Mode:	AND	Max Results:	100

Event#: 3 MS(E-) Ret. Time : 8.410 Scan# : 1413

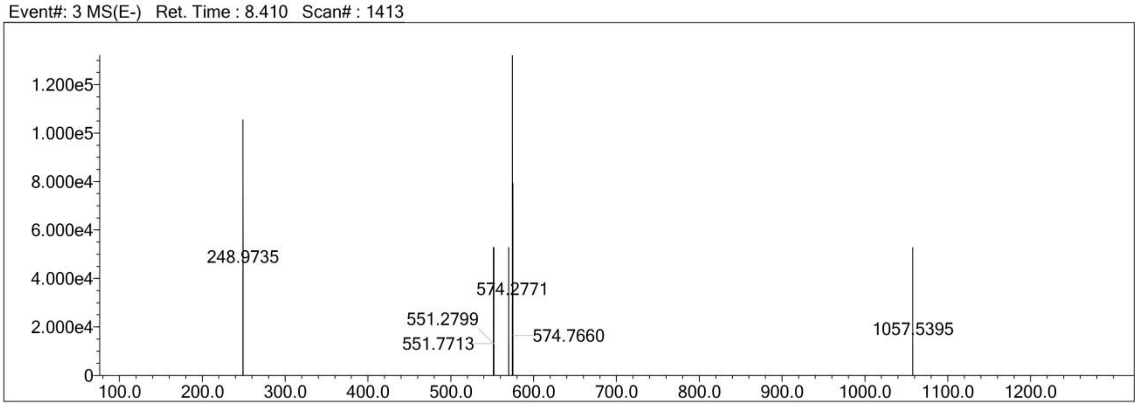

Measured region for 1057.5395 m/z

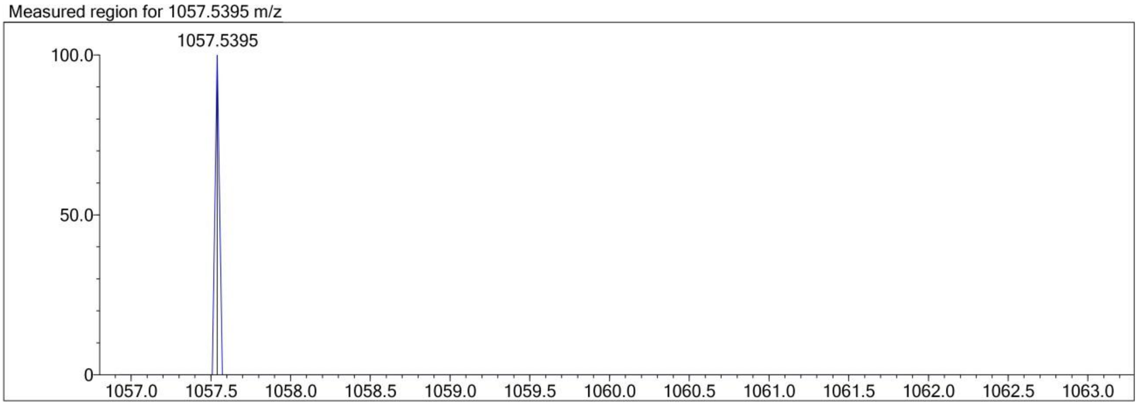

C53 H86 O21 [M-H]- : Predicted region for 1057.5589 m/z

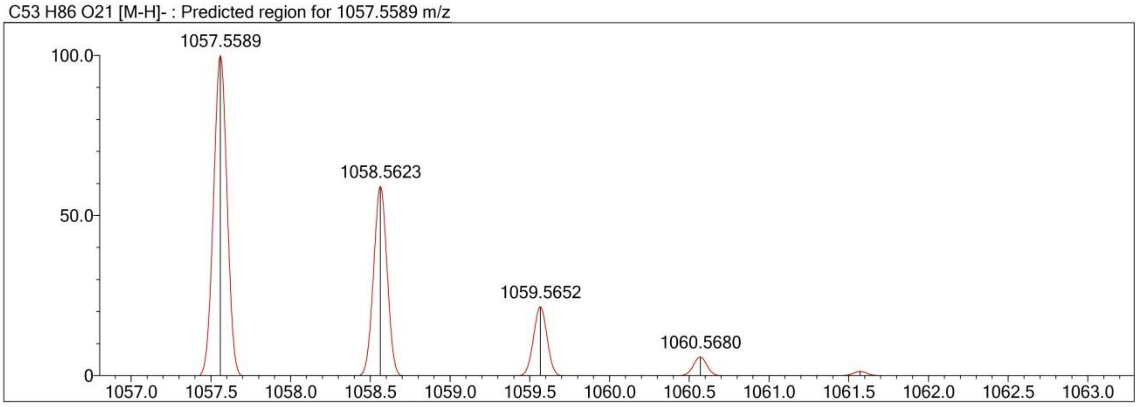

Rank	Score	Formula (M)	Ion	Meas. m/z	Pred. m/z	Df. (mDa)	Df. (ppm)	Iso	DBE
25	0.00	C53 H86 O21	[M-H]-	1057.5395	1057.5589	-19.4	-18.34	0.00	11.0

Figure S23 HR-ESI-MS spectrum of **6**

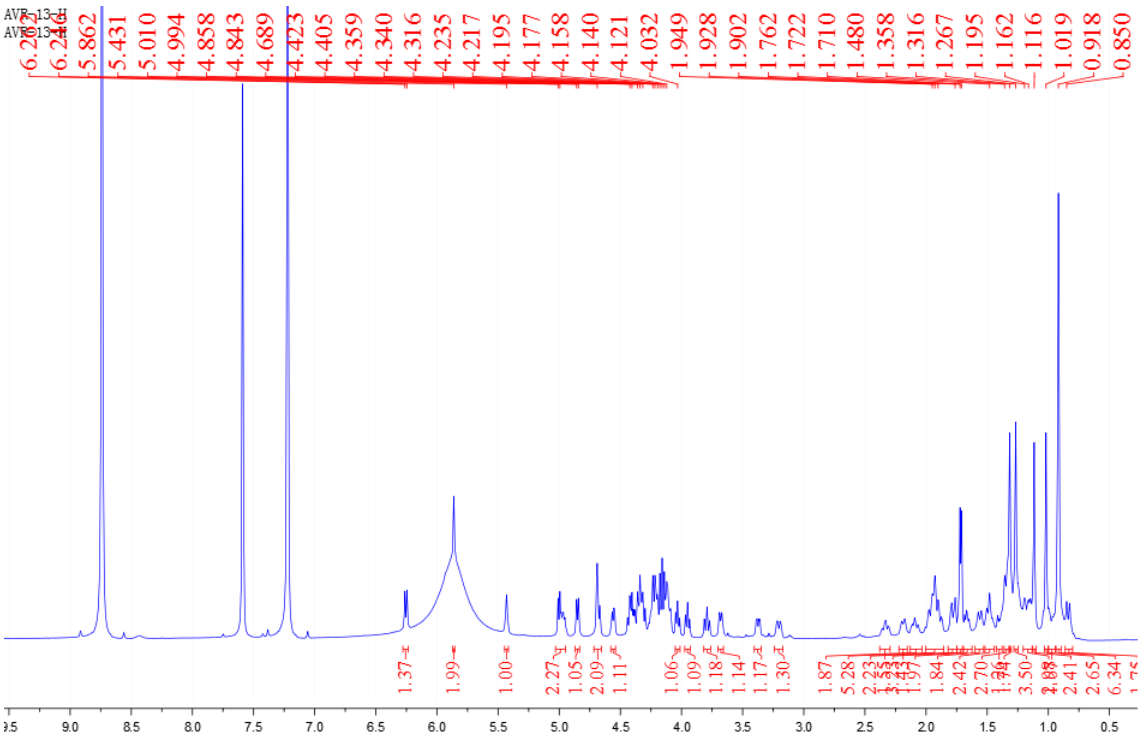

Figure S24 ^{1}H NMR spectrum of **6** in Pyridine-d_5 (500 MHz)

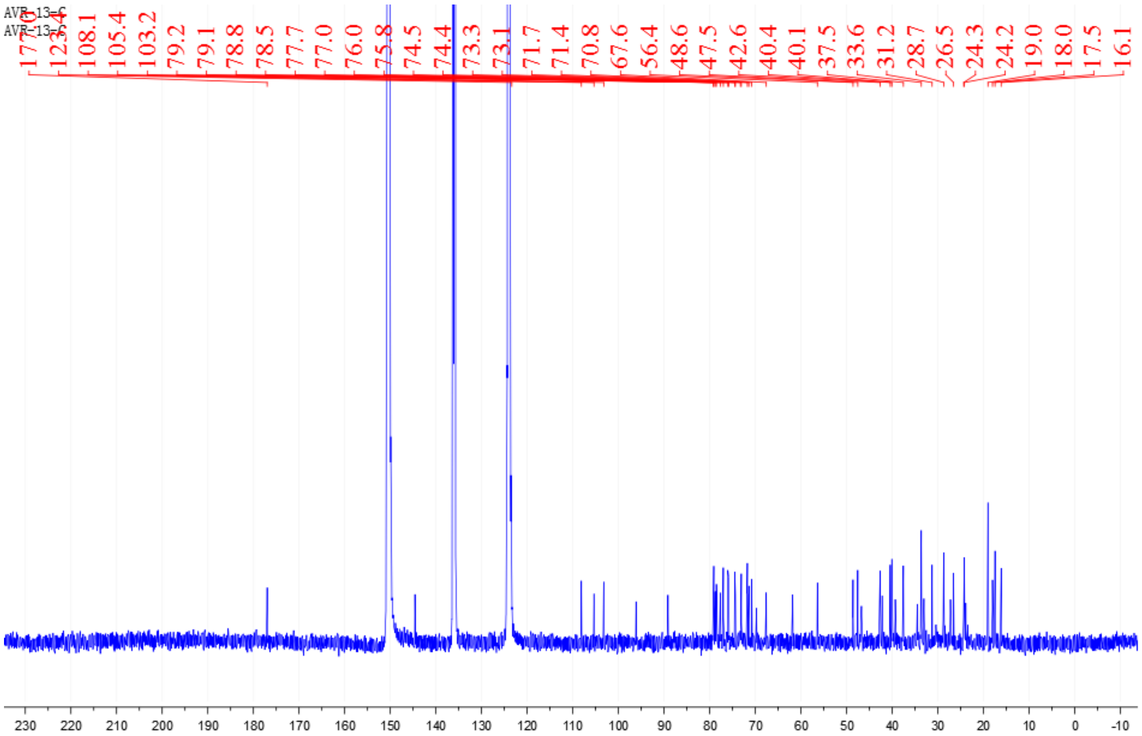

Figure S25 ^{13}C NMR spectrum of **6** in Pyridine-d_5 (125 MHz)

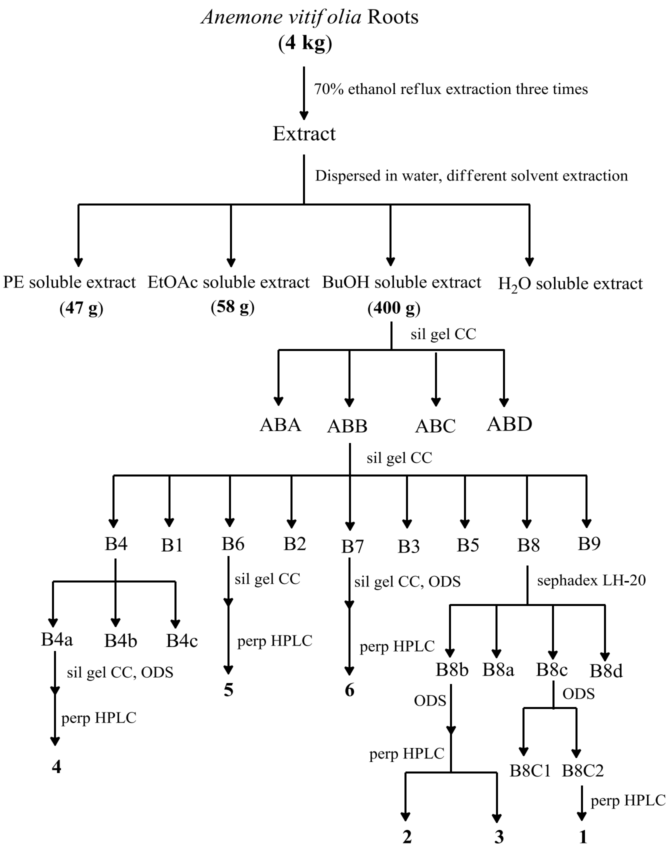

Figure S26 The separation of the compounds 1-6
